# Exploiting photosynthesis-driven P450 activity to produce indican in tobacco chloroplasts

**DOI:** 10.3389/fpls.2022.1049177

**Published:** 2023-01-09

**Authors:** Silas B. Mellor, James B. Y. H. Behrendorff, Johan Ø. Ipsen, Christoph Crocoll, Tomas Laursen, Elizabeth M. J. Gillam, Mathias Pribil

**Affiliations:** ^1^ Section for Plant Biochemistry, Department of Plant and Environmental Science, University of Copenhagen, Frederiksberg, Denmark; ^2^ School of Biology and Environmental Science, Queensland University of Technology, Brisbane, QLD, Australia; ^3^ Australian Research Council (ARC) Centre of Excellence in Synthetic Biology, Queensland University of Technology, Brisbane, QLD, Australia; ^4^ Section for Forest, Nature and Biomass, Department of Geosciences and Natural Resource Management, University of Copenhagen, Frederiksberg, Denmark; ^5^ DynaMo Center, Section for Molecular Plant Biology, Department of Plant and Environmental Science, University of Copenhagen, Frederiksberg, Denmark; ^6^ School of Chemistry and Molecular Biosciences, University of Queensland, Brisbane, QLD, Australia; ^7^ Section for Molecular Plant Biology, Department of Plant and Environmental Science, University of Copenhagen, Frederiksberg, Denmark

**Keywords:** cytochrome P450, chloroplast, indigo, transient expression, photosynthesis, ferredoxin, metabolic engineering

## Abstract

Photosynthetic organelles offer attractive features for engineering small molecule bioproduction by their ability to convert solar energy into chemical energy required for metabolism. The possibility to couple biochemical production directly to photosynthetic assimilation as a source of energy and substrates has intrigued metabolic engineers. Specifically, the chemical diversity found in plants often relies on cytochrome P450-mediated hydroxylations that depend on reductant supply for catalysis and which often lead to metabolic bottlenecks for heterologous production of complex molecules. By directing P450 enzymes to plant chloroplasts one can elegantly deal with such redox prerequisites. In this study, we explore the capacity of the plant photosynthetic machinery to drive P450-dependent formation of the indigo precursor indoxyl-β-D-glucoside (indican) by targeting an engineered indican biosynthetic pathway to tobacco (*Nicotiana benthamiana*) chloroplasts. We show that both native and engineered variants belonging to the human CYP2 family are catalytically active in chloroplasts when driven by photosynthetic reducing power and optimize construct designs to improve productivity. However, while increasing supply of tryptophan leads to an increase in indole accumulation, it does not improve indican productivity, suggesting that P450 activity limits overall productivity. Co-expression of different redox partners also does not improve productivity, indicating that supply of reducing power is not a bottleneck. Finally, *in vitro* kinetic measurements showed that the different redox partners were efficiently reduced by photosystem I but plant ferredoxin provided the highest light-dependent P450 activity. This study demonstrates the inherent ability of photosynthesis to support P450-dependent metabolic pathways. Plants and photosynthetic microbes are therefore uniquely suited for engineering P450-dependent metabolic pathways regardless of enzyme origin. Our findings have implications for metabolic engineering in photosynthetic hosts for production of high-value chemicals or drug metabolites for pharmacological studies.

## Introduction

Biotechnological production of valuable and useful molecules is a burgeoning field, and in some cases has become the preferred method to produce biopharmaceuticals or small molecule compounds (Madhavan et al., 2021; Wu et al., 2021). Bio-based small molecule production has many environmental advantages by virtue of being carried out in aqueous systems using simple sugar or organic acid precursor molecules. This renders both feedstock and waste streams less environmentally harmful and, where extraction from natural sources is otherwise required, avoids putting undue stress on scarce or endangered natural resources. A growing number of production processes relying on metabolically engineered organism is emerging as consumer demand and interest for products from sustainable sources increases (Nielsen et al., 2022). Microbial hosts still dominate the field of engineered biomolecule production, both for protein- and small molecule-based therapeutics, but plants can be economically competitive vis-à-vis microorganisms for heterologous production (Holtz et al., 2015). Plants also offer the enticing possibility of coupling biochemical production directly to the assimilation of light energy and carbon *via* photosynthesis; truly green chemistry (Sørensen et al., 2022). Recent thorough techno-economic analyses have shown that bio-fuel manufacturing in photosynthetic algae can be economically viable given optimal environmental conditions, especially if co-products such as protein can be extracted in parallel from the same strain (Roles et al., 2021; Karan et al., 2022). This offers hope that with proper design and optimization, production of high value molecules through metabolic engineering in plants could prove economically feasible in the future.

Most examples of plants engineered to produce small molecules have introduced pathways in the cytosol, but compartmentation of pathways is a growing trend (Huttanus and Feng, 2017), and follows an increased understanding of the compartmentalized nature of many biosynthetic pathways (Heinig et al., 2013). Chloroplasts serve as hubs for cellular bioenergy generation by virtue of their primary photosynthetic processes and their role as a carbon fixation, storage and re-mobilization compartment. Chloroplasts are also highly diverse metabolic centers that supply precursors for a wide variety of central and specialized metabolic processes (Heinig et al., 2013; Nielsen et al., 2016). As such they are attractive compartments for introducing a variety of metabolic pathways, such as those dependent on oxyfunctionalization by cytochrome P450 enzymes (Mellor et al., 2016). Because of the widespread occurrence of P450s throughout all kingdoms of life, their prominent roles in specialized metabolism and ability to catalyze remarkably diverse chemical transformations (Podust and Sherman, 2012; Liu et al., 2020; Behrendorff, 2021), they are important enzymes in the metabolic engineering toolbox. Most P450s in eukaryotes localize to the endoplasmic reticulum (ER) where they are anchored by a single N-terminal transmembrane domain. Eukaryotes likewise harbor distinct diflavin cytochrome P450 reductases (CPRs), which are also membrane bound and localize to the ER confining metabolism to the two-dimensional membrane lattice (Laursen et al., 2021). Being membrane proteins and dependent on dedicated reductase systems, P450s are often difficult to functionally reconstitute in heterologous hosts and may require significant engineering effort to reach acceptable functionality (Renault et al., 2014; Liu et al., 2020; Jensen et al., 2021).

**Figure 1:**
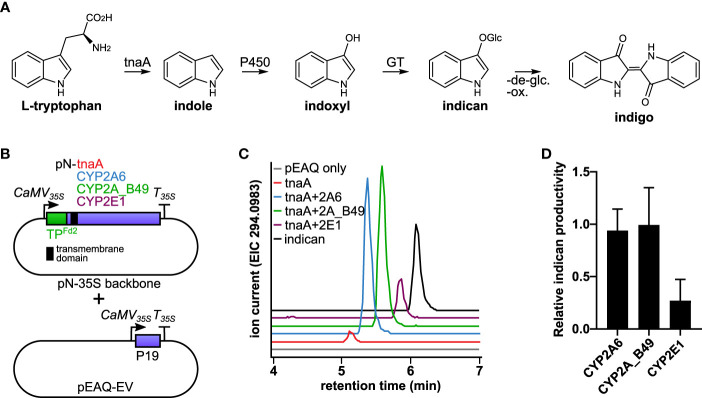
**(A)** Diagram of the indigo pathway introduced into tobacco chloroplasts in this study. Tryptophan is converted by tnaA to indole, which is hydroxylated to indoxyl by P450s and subsequently glycosylated by endogenous tobacco glucosyltransferases (GT) to form indican. Indigo can be formed from indican by de-glycosylation and oxidative polymerization. **(B)** Schematic of constructs used to establish indican production in chloroplasts using human CYP2 isoforms. Both tnaA and P450s were targeted to chloroplast by *Arabidopsis* Fd2 transit peptide (TP^Fd2^). The P450s inserted into thylakoid membranes by their N-terminal transmembrane (indicated by a black square). *Agrobacterium* carrying vector encoding tnaA were infiltrated on its own or in combination with vector carrying a human CYP2 isoform, as indicated at equal ODs together with empty pEAQ vector (pEAQ-EV) to deliver the P19 suppressor of silencing protein. **(C)** Production of indican by CYP2A6, CYP2A_B49, and CYP2E1. Extracted ion chromatograms from LC-QTOF-MS/MS analysis of the [M-H]^-^ molecular ion of indican (*m/z* 294.0983). **(D)** Relative productivity of CYP2A6, CYP2A_B49 and CYP2E1 co-expressed transiently in tobacco together with *E. coli* tnaA, determined by comparison of internal standard-normalized indican peak areas from 10-11 individually infiltrated tobacco leaves (error bars show SD).

We and others have shown heterologous targeting of P450s to tobacco chloroplasts by fusion to the chloroplast targeting peptide from *Arabidopsis* ferredoxin 2 and found that photosynthesis supplies reducing equivalents to the P450s *via* interaction with chloroplast ferredoxins (Nielsen et al., 2013; Mellor et al., 2016). Targeting P450 enzymes to thylakoid membranes and avoiding the need for co-expression of a dedicated reductase is an advantage for engineering of P450-dependent pathways but has only been shown for a handful of P450s to date (Nielsen et al., 2013; Xue et al., 2014; Gnanasekaran et al., 2015; Berepiki et al., 2016; Fräbel et al., 2018; Li et al., 2019). Several human P450s produce the insoluble blue dye indigo upon recombinant expression in *E. coli* (Gillam et al., 1999). Expression of the indole hydroxylase CYP2A6 in tobacco together with either maize indole-3-glycerol phosphate lyase (BX1) or *E. coli* tryptophanase (tnaA) results in accumulation of the glucosylated indigo precursor indoxyl β-D-glucoside (**Figure 1A**, indican) (Warzecha et al., 2007; Fräbel et al., 2018). Here we test the ability of tobacco chloroplasts to support human P450s by targeting three indole hydroxylating P450 isoforms and tnaA to tobacco chloroplasts and use the system to explore strategies for optimizing photosynthesis-driven indican productivity. We find that maximal productivity is achieved by expressing P450 and tnaA from the same vector and co-express a feedback insensitive mutant of 3-deoxy-D-arabino-heptulosonate 7-phosphate synthase (AroG*), which catalyzes the first committed step of the shikimate pathway to boost the supply of tryptophan. Its transient expression in tobacco yields 37 to 115-fold increases in aromatic amino acid pools, with concurrent effects on indole production by tryptophanase. We also co-express different redox partners but find that neither these nor AroG* affect indican productivity. Through *in vitro* study on isolated electron transfer proteins, we find that plant ferredoxin outcompetes other redox partners in coupling photosynthetic electron transport and P450 activity. Together, our results show that P450 activity constitutes the major bottleneck for overall indican productivity in our transient expression system.

## Materials and methods

### Expression vectors for *E. coli* expression

The pTrc99a-AtFd1 vector was a kind gift from Guy T. Hanke. Flavodoxin genes were amplified by PCR with overhangs and cloned into pET14b vector using NEB HiFi DNA assembly master mix (New England Biolabs). The IsiB gene from *Synechocystis* sp. PCC6803 was amplified by colony PCR. The flavodoxin-like domain of human CPR (residues 62-241, referred to as HsCPR^62-241^) was amplified from the bicistronic expression vector pCWori/CYP2A_B49/hNPR (Strohmaier et al., 2019). After assembly, colony PCR positive clones were confirmed by sequencing.

### Expression vectors for transient expression in *N. benthamiana*

The CYP2A_B49 variant was selected based on indigo productivity in *E. coli* generated from a DNA shuffling library using CYP2A5, CYP2A6, and CYP2A13 isoforms (Behrendorff et al., 2013; Strohmaier et al., 2019). Cytochrome P450 genes were amplified from *E. coli* expression constructs (Gillam et al., 1999). TnaA and AroG* genes were synthesized by Twist Bioscience (San Francisco, USA). All inserts were PCR amplified with overhangs and were cloned into the pN vector (Karimi et al., 2002; Behrendorff et al., 2019) using NEB HiFi DNA assembly master mix (New England Biolabs). For bidirectional promoter constructs, the pN vector was used without 35S promoter and insert cassette. AtUBQ10 promoter was amplified from a previously reported construct (Behrendorff et al., 2019). *Nicotiana tabacum* ferredoxin chloroplast transit peptide and *Arabidopsis thaliana* Hsp18.2 terminator were synthesized by Twist Bioscience (San Francisco, USA). The Ubi.U4 promoter was amplified from *N. tabacum* genomic DNA. After assembly, colony PCR positive clones were confirmed by sequencing. pEAQ-HT vectors with AroG*, AtFd1, IsiB^6803^, HsCPR^62-241^ and HsCPR^fl^ inserts were synthesized by Twist Bioscience (San Francisco, USA). Nucleotide sequences of the genes, promoters and terminators used are given in the supplementary materials (**Supplementary Table S1**).

### Transient expression in *N. benthamiana*

Colonies of *Agrobacterium tumefaciens* GV3101 transformed with vectors of interest were inoculated in YEP in the presence of 10 µg mL^-1^ rifampicin, 25 µg mL^-1^ gentamycin with 100 µg mL^-1^ spectinomycin (pN, pBJ1) or 50 µg mL^-1^ kanamycin (pEAQ) and grown with shaking (200 rpm) for 2 d at 28°C. Cells were harvested and suspended in infiltration buffer (10 mM MES pH 5.6, 10 mM MgCl_2_, 200 µM acetosyringone) to the desired OD and incubated at RT with gentle agitation for 2 h. Leaves of 4-6 week old greenhouse-grown tobacco plants were infiltrated on the abaxial leaf surface. Plants were allowed to dry before returning to greenhouse.

### Untargeted LC-QTOF-MS/MS

Samples (80-120 mg) were taken from *N. benthamiana* plants 3-5 days post inoculation (dpi), snap frozen in liquid N_2_ and homogenized using a Retsch Tissue-Lyzer mixer mill (2-4x30 s, 20 s^-1^). The homogenized material was extracted with 0.5 mL 80% methanol for 30 min at 4°C. Samples were centrifuged (2,250 g, 10 min, 4°C) and supernatant diluted 4-fold into milliQ water containing 1 mg L^-1^ Leu-enkephalin as internal standard. LC-MS/MS was performed on a Dionex UltiMate 3000 Quaternary Rapid Separation UHPLC+ focused system (Thermo Fisher Scientific, Germering, Germany) using a Kinetex XB-C18 column (100 × 2.1 mm, 1.7 μm, 100 Å, Phenomenex). Formic acid (0.05% v/v) in water and acetonitrile (supplied with 0.05% v/v formic acid) were employed as mobile phases A and B, respectively. Gradient conditions were as follows: 0.0-0.5 min 5% B; 0.5−.0 17.5 min 5−45% B, 17.5-23.0 min 45-75% B, 23.0-25.0 min 75-100% B, 25.0−26.95 min 100% B, 26.95-27.0 min 100−5% B, and 27.0−30.0 min 5% B. The flow rate of the mobile phase was 300 μL min^-1^. The column temperature was maintained at 30°C. UV spectra for each sample were acquired at 214, 260, 280, and 300 nm. The UHPLC was coupled to a Compact micrOTOF-Q mass spectrometer (Bruker, Bremen, Germany) equipped with an electrospray ion source (ESI) operated in positive or negative ion mode. The ion spray voltage was maintained at +4500 V and -3900 V in positive and negative ion mode, respectively. Dry temperature was set to 250°C, and nitrogen was used as the dry gas (8 L min^-1^), nebulizing gas (2.5 bar), and collision gas in both ion modes. Collision energy was set to 10 eV and 15 eV in positive and negative ion mode, respectively. MS spectra were acquired with a sampling rate of 2 Hz in an *m/z* range from 50 to 1000 and MS/MS spectra in a range of *m/z* 100-800 in positive ion mode while in negative ion mode MS spectra were acquired with a sampling rate of 3 Hz in an *m/z* range from 50 to 1400 and MS/MS spectra in a *m/z* range from 100-1000. Na-formate clusters were used for mass calibration in both ion modes. Positive ion mode was used for untargeted metabolomics and negative ion mode was used to detect indican. Indole did not provide detectable MS signal and was instead quantified using its UV absorbance at 214 nm against a standard curve containing 0.75-100 µM indole in 20% methanol.

### Analysis of untargeted MS data

Data analysis was performed using MS-DIAL (Lai et al., 2017). A MS1 tolerance of 0.05 Da was used, and features were matched to the positive ionization LC-MS/MS library from Mass Bank of North America (https://mona.fiehnlab.ucdavis.edu/) using MS1 and MS2 tolerances of 0.01 Da and 0.025 Da, respectively. After alignment, data was normalized to the peak area of the Leu-Enk internal standard. PCA analysis was performed on log_10_-transformed and auto-scaled data using only reference matched features. Normalized peak areas of compounds of interest were extracted directly from MS-DIAL and plotted using GraphPad Prism.

### Quantification of indican by LC-QqQ-MS/MS

Samples were collected at 5 dpi, homogenized and extracted as for untargeted MS analysis. Extracts were diluted 20 times into 20% methanol with 50 µg L^-1^ *p*-hydroxybenzaldehyde as internal standard and subjected to LC-MS analysis. Chromatography was performed on a 1290 Infinity II UHPLC system (Agilent Technologies), using a Kinetex XB-C18 column (100 x 2.1 mm, 1.7 µm, 100 Å, Phenomenex, Torrance, CA, USA). Formic acid (0.05%, v/v) in water and acetonitrile (supplied with 0.05% v/v formic acid) were employed as mobile phases A and B, respectively. The elution profile for indican was: 0.0-2.5 min, 5-65% B; 2.5-3.0 min 65-100% B, 3.0-4.0 min 100% B, 4.0-4.1 min, 100-5% B and 4.1-5.0 min 5% B. The mobile phase flow rate was 400 µL min^-1^. The column temperature was maintained at 40°C. The liquid chromatography was coupled to an Ultivo Triplequadrupole mass spectrometer (Agilent Technologies) equipped with a Jetstream electrospray ion source (ESI) operated in negative ion mode. The ion spray voltage was set to -3000 V. Dry gas temperature was set to 325°C and dry gas flow to 9 L min^-1^. Sheath gas temperature was set to 325°C and sheath gas flow to 12 L min^-1^. Nebulizing gas was set to 40 psi. Nitrogen was used as dry gas, nebulizing gas and collision gas. Multiple reaction monitoring (MRM) was used to monitor precursor ion → fragment ion transitions (**Supplementary Table S2**). MRM transitions and instrument parameters were optimized using reference standards. Both Q1 and Q3 quadrupoles were maintained at unit resolution. Mass Hunter Quantitation Analysis for QqQ software (Version 10.1, Agilent Technologies) was used for data processing. Indican peak areas were converted to concentrations by comparing against a standard curve ranging from 0.025-5 µM. Standard curves of authentic indican (abcam) were prepared in blank tobacco matrix prepared as described above from untransformed *N. benthamiana* to account for matrix effects.

### Quantification of amino acids by LC-QqQ-MS/MS

Samples were collected 5 dpi as described above but extracted using 85% methanol. Subsequently, samples were mixed 1:10 (v/v) with ^13^C, ^15^N labeled amino acids (Algal amino acids ^13^C, ^15^N, Isotec, Miamisburg, US) at a concentration of 10 µg*mL^-1^ either directly or after 10-fold dilution with water and analyzed by LC-MS. The analysis was performed as described in (Mirza et al., 2016) with changes as detailed below. Chromatography was performed on an Advance UHPLC system (Bruker, Bremen, Germany) using a Zorbax Eclipse XDB-C18 column (100 x 3.0 mm, 1.8 µm, Agilent Technologies, Germany). Formic acid (0.05% v/v) in water and acetonitrile (supplied with 0.05% v/v formic acid) were employed as mobile phases A and B, respectively. The elution profile was: 0-1.2 min 3% B; 1.2-4.3 min 3-65% B; 4.3-4.4 min 65-100% B; 4.4-4.9 min 100% B, 4.9-5.0 min 100-3% B and 5.0-6.0 min 3% B. Mobile phase flow rate was 500 µL min^-1^ and column temperature was maintained at 40°C. The liquid chromatography was coupled to an EVOQ Elite TripleQuad mass spectrometer (Bruker, Bremen, Germany) equipped with an electrospray ionization source (ESI). Instrument parameters were optimized by infusion experiments with pure standards. The ion spray voltage was maintained at 3000 V in positive ion mode. Cone temperature was set to 300°C and cone gas flow to 20 psi. Heated probe temperature was set to 400°C and probe gas flow set to 50 psi. Nebulizing gas was set to 60 psi and collision gas to 1.6 mTorr. Nitrogen was used as both cone gas and nebulizing gas and argon as collision gas. Analyte precursor ion → fragment ion transitions were monitored by MRM, with transitions chosen as described by Jander et al., with additions from Docimo et al. for Arg and Lys (Jander et al., 2004; Docimo et al., 2012). Both Q1 and Q3 quadrupoles were maintained at unit resolution. Bruker MS Workstation software (Version 8.2.1, Bruker, Bremen, Germany) was used for data acquisition and processing. Individual amino acids in the sample were quantified by comparison of peak areas of light- amino acids with ^13^C, ^15^N-labeled amino acid internal standards of known concentrations, except for tryptophan, asparagine and glutamine. Tryptophan was quantified using ^13^C, ^15^N-Phe applying a response factor of 0.42, asparagine and glutamine were quantified using ^13^C, ^15^N-Asp and ^13^C, ^15^N-Glu, respectively, applying a response factor of 1.0 (Docimo et al., 2012).

### Overexpression and purification of electron transfer proteins

AtFd1 was expressed in *E. coli* DH5α after inducing cells with 1 mM Isopropyl *β-D*-1-thiogalactopyranoside (IPTG) at OD=1.0 and growing the cells overnight at 37°C with 200 rpm shaking. Purification was performed essentially as previously described (Matsumura et al., 1999). Flavodoxins were expressed in *E. coli* BL21 (DE3) after inducing cells with 1 mM IPTG at OD=0.5-0.6 and growing the cells overnight at 30°C (IsiB^6803^) or 25°C (HsCPR^62-241^). Cells were and harvested by centrifugation (10,000 g for 30 min at 4°C) and stored at -20°C. Cells were lysed by incubation for 30 min on ice with stirring in lysis buffer (50 mM Tris-HCl pH 7.5, 300 mM NaCl, 5 mM imidazole, 0.5 mg mL^-1^ lysozyme) before sonicating for 15 cycles (30 s, 50% duty cycle, amplitude 6 followed by 1 min cooling period) using a Branson Sonifier 450 sonicator. Lysates were clarified by centrifugation (14,000 g, 20 min, 4°C) followed by filtering at 0.45 µm. Purification was performed using a Cytiva HisTrap HP column at 1 mL min^-1^ on an ÄKTA Start purification system (Cytiva). The column was washed (20 column volumes, 50 mM Tris-HCl pH 7.5, 300 mM NaCl, 5 mM imidazole) prior to eluting with a linear gradient of 5-250 mM imidazole over 30 column volumes. Yellow fractions were pooled, desalted into 50 mM Tris-HCl (pH 7.5) and checked for purity using SDS-PAGE.

### Reconstitution of flavodoxins and determination of FMN extinction coefficients

Flavodoxins (~1-2 mM) were reconstituted with 10 mM FMN on ice overnight and desalted into 50 mM tris-HCl (pH 7.5). FMN incorporation was estimated by comparing with calculated absorbance ratios for FMN and protein at 280 nm (0.166, and 0.210, respectively for IsiB^6803^ and HsCPR^62-241^). FMN incorporation was typically >95%. Flavodoxin extinction coefficients were determined by adapting established protocols (Mayhew and Massey, 1969) for microtiter plates. FMN cofactor was extracted by successive treatments with 5% TCA on ice, the supernatants recovered after centrifugation at 10,000 g for 10 min and neutralized by adding K_2_HPO_4_ to 0.3 M. FMN extinction coefficients were calculated as ϵ = 12.2 mM^-1^ cm^-1^ * (A_FMN_^bound^/A_FMN_^free^), based on spectrophotometric measurements of FMN extracted from flavodoxins and holoflavodoxins measured in UV-clear microtiter plates using a BioTek Synergy H1 plate reader and corrected for buffer background. Coefficients of 8.7 and 10.1 mM^-1^ cm^-1^ were obtained, respectively, for IsiB^6803^ and HsCPR^62-241^.

### Isothermal titration calorimetry

Isothermal titration calorimetry was performed on a MicroCal PEAQ-ITC calorimeter (Malvern Panalytical). Titrations were performed by injecting FMN (>95% purity, Merck Sigma-Aldritch) into a solution of pure IsiB^6803^ or HsCPR^62-241^ apoprotein desalted into 50 mM Tris-HCl pH 7.5. The same preparation of buffer was used for both protein and ligand. Protein concentration in the cell was 15 µM and titrant used was 150 µM. After a priming injection of 0.4 µL titration was performed using a total of 12 injections of 3 µL and the resulting data was fitted using the MicroCal PEAQ-ITC analysis software (Malvern Panalytical).

### Thylakoid preparation

Thylakoids were prepared by homogenizing leaves of dark-adapted tobacco plants thoroughly in ice-cold homogenization buffer (0.4 M sucrose, 20 mM Tricine-NaOH pH 7.5, 10 mM NaCl, 5 mM MgCl_2_, 100 mM sodium ascorbate, 5 mg mL^-1^ bovine serum albumin) using a blender. The homogenate was filtered centrifuged for 10 min at 5,000 g, 4°C. Pelleted chloroplasts were resuspended in 5 mM Tricine pH 7.9 and left to rupture osmotically on ice for 15 min. Thylakoid membranes were pelleted at 11,200 g, 4°C for 10 min and resuspended in storage buffer (0.4 M sucrose, 20 mM Tricine-NaOH pH 7.5, 10 mM NaCl, 5 mM MgCl_2_, 20% v/v glycerol). Chlorophyll concentration of preparations was measured according to Lichtenthaler (Lichtenthaler, 1987).

### Light-dependent coumarin 7-hydroxylation assays

The activity of CYP2A_B49 was measured after isolating thylakoid membranes from pN-CYP2A_B49-infiltrated tobacco plants. Assays contained 50 mM tricine (pH 7.5), 100 mM NaCl, 10 µM coumarin, CYP2A_B49-containing thylakoids (100 µg chl mL^-1^) and redox partners (0.1-30 µM). Reactions were maintained at 25°C and were started by illuminating assays in black 96-well microtiter plate using a rectangular LED array connected to a laboratory power supply and adjusted to deliver a photon flux of 170 µmol m^-2^ s^-1^ light intensity. Reactions were stopped after 2-10 min by addition of 0.33 µM methyl viologen and switching off the light. Plates were centrifuged (2,850 g, 5 min) and supernatants were filtered through 0.45 µm filters to remove thylakoid membranes. Umbelliferone fluorescence was measured against authentic standards (5-100 nM) dissolved in 50 mM tricine (pH 7.5), using excitation and emission wavelengths of 370 and 450 nm, respectively. Enzymatic rates were corrected for background rates without redox partner and the data fitted to the Michaelis-Menten equation to obtain kinetic parameters using GraphPad Prism.

### *Cytochrome c* reduction assays

Light-dependent cytochrome *c* reduction was measured using a Shimadzu UV-2550 double beam spectrophotometer fitted with a light fiber and 550 nm band pass filters on the measuring beam windows. Temperature was controlled at 25°C by thermostat. Assays contained 50 mM Tricine (pH 7.5), 100 mM NaCl, 200 µM cytochrome *c*, thylakoids (10 µg chl mL^-1^) and 0.01-4 µM redox partners. The assay was initiated by switching on the light source (Schott KL-1500 fitted with red-light bandpass filters) and the activity was followed for 60 s. Initial rates of cytochrome *c* reduction were calculated by taking a reduced-oxidized extinction coefficient of 18.5 mM^-1^ cm^-1^ (Kubota et al., 1992), corrected for the rate of cytochrome *c* reduction without redox partner present and fitted to the Michaelis-Menten equation using GraphPad Prism.

### Ferredoxin : NADP+ reductase assays

Assays were carried out at RT in 96-well microtiter plates by adding 50 mM Tris-HCl (pH 7.5), 100 mM NaCl, 1 mM MgCl_2_, 5 mM glucose 6-phosphate, 5 U mL^-1^ glucose 6-phosphate dehydrogenase, 200 µM cytochrome *c*, and 40 nM spinach FNR to microtiter wells containing NADPH (500 µM final) and electron carriers (1-100 µM final). Absorbance was measured at 550 nm for 2 min using a BioTek Syntergy H1 plate reader. Initial rates of cytochrome *c* reduction were calculated taking a reduced-oxidized extinction coefficient of 18.5 mM^-1^ cm^-1^ (Kubota et al., 1992) and corrected for the background cytochrome *c* reduction rate in assays without electron carriers. The data was fitted to the Michaelis-Menten equation using GraphPad Prism.

## Results

### Photosynthesis-driven indican biosynthesis using human cytochrome P450 enzymes

Chloroplasts were previously shown to provide membrane insertion and light-dependent supply of reducing power towards catalysis by plant cytochrome P450 enzymes (Jensen et al., 2011; Nielsen et al., 2013). We wanted to investigate whether this principle could be extended to support catalytic activity by distantly related P450 enzymes such as those from animals. We therefore expressed human P450s CYP2A6 and CYP2E1 and the engineered variant CYP2A_B49, all of which hydroxylate indole to indoxyl (Gillam et al., 1999) transiently targeted to chloroplasts in *Nicotiana benthamiana*, and used the hydroxylation of indole as a readout for P450 activity (**Figure 1A**). Indole hydroxylation was detectable as accumulation of indoxyl β-D-glucoside, the major hydroxylated indole metabolite that occurs in plants as a result of indoxyl glycosylation by endogenous plant glucosyltransferases (Warzecha et al., 2007). Since tryptophan biosynthesis occurs in chloroplasts, we co-expressed *E. coli* L*-*tryptophan indole lyase (tnaA), which hydrolyses tryptophan to indole, also with chloroplast targeting to provide indole precursor for the P450s. We used the chloroplast transit peptide from *Arabidopsis* ferredoxin 2 (TP^Fd2^), which can direct both membrane targeted P450s and soluble enzymes to chloroplasts in tobacco (Nielsen et al., 2013), fused at the N-terminus of both P450s and tnaA to target the enzymes to chloroplasts (**Figure 1B**). *Agrobacterium* strain transfected with empty pEAQ-HT vector (Peyret and Lomonossoff, 2013) was mixed with pN-carrying strains at equal ODs in all transfections to reduce silencing and ensure consistent maximal expression of tnaA and P450s. Expression of tnaA alone led to detectable indole accumulation in leaf samples (**Supplementary Figure S1**). We detected trace amounts of indican when tnaA was expressed alone, possibly due to presence of endogenous indole-metabolizing enzymes, as reported previously (Warzecha et al., 2007). However accumulation was considerably higher when any P450 was co-expressed with tnaA (**Figure 1C**). Four additional unknown compounds were detected upon expression of tnaA with CYP2A6 or CYP2A_B49 (**Supplementary Figure S2A**). Three of them had identical parent masses and were consistent with dioxyindole glucosides (**Supplementary Figure S2B**), while the parent mass of the fourth compound was consistent with hydroxylated indole acetic acid glucoside (**Supplementary Figure S2C**). Co-infiltration of tnaA with CYP2A6 and CYP2A_B49 yielded similar indican accumulation (**Figure 1D**), and CYP2A_B49 was used for subsequent experiments.

### Optimization of transient indican production by T-DNA engineering

**Figure 2:**
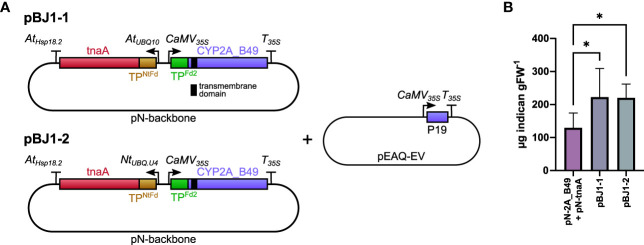
**(A)** Schematic of pBJ bidirectional promoter T-DNA constructs used in this experiment. The transmembrane domain of CYP2A_B49 is indicated by a black bar. Co-expression tnaA and CYP2A_B49 was done either by co-infiltrating pN-tnaA and pN-CYP2A_B49 vectors or by infiltrating the bi-directional T-DNA pBJ1-1 or pBJ1-2 vectors at equal ODs together with pEAQ-EV to supply the P19 suppressor of silencing gene. **(B)** Indican accumulation resulting from expressing tnaA and CYP2A_B49 from individual pN vectors or together from pBJ1-1 or pBJ1-2 vectors. Bars show averages from 6 individually infiltrated tobacco leaves of similar age with error bars showing standard deviation. Asterisks indicate statistically significant differences (*p < 0.05*) in *post-hoc* pairwise Holm-Šídák tests following one-way ANOVA.

Indican production requires both tnaA and P450 transgenes and we hypothesized that placing both transgenes on the same T-DNA would improve indican productivity by ensuring all transfected cells receive both transgenes. To test this hypothesis, we cloned two bi-directional pN constructs (pBJ1-1 and pBJ1-2) that contained both tnaA and CYP2A_B49 genes driven by different promoters (**Figure 2A**). The CYP2A_B49 gene was driven by the same 35S promoter/terminator combination as our single gene pN vectors in both pBJ1 versions, whereas tnaA expression was driven either by the *Arabidopsis* polyubiquitin 10 promoter (At_UBQ10_, pBJ1-1) or *N. tabacum* polyubiquitin U4 promoter (Kang et al., 2003) (Nt_UBQ.U4_, pBJ1-2), both with the *Arabidopsis* Hsp18.2 terminator (Nagaya et al., 2010). We also used *N. tabacum* ferredoxin CTP to avoid re-using that of *Arabidopsis* Fd2 to target tnaA to chloroplasts. Having both enzymes on the same T-DNA nearly doubled indican accumulation compared to mixing individual *Agrobacterium* strains, with the pBJ1-1 and pBJ1-2 constructs producing equivalent indican amounts (**Figure 2B**). These results indicate that expressing multiple interdependent transgenes from the same T-DNA can increase productivity compared to introducing the transgenes on individual vectors.

### Engineering the shikimate pathway to boost aromatic amino acid accumulation

**Figure 3:**
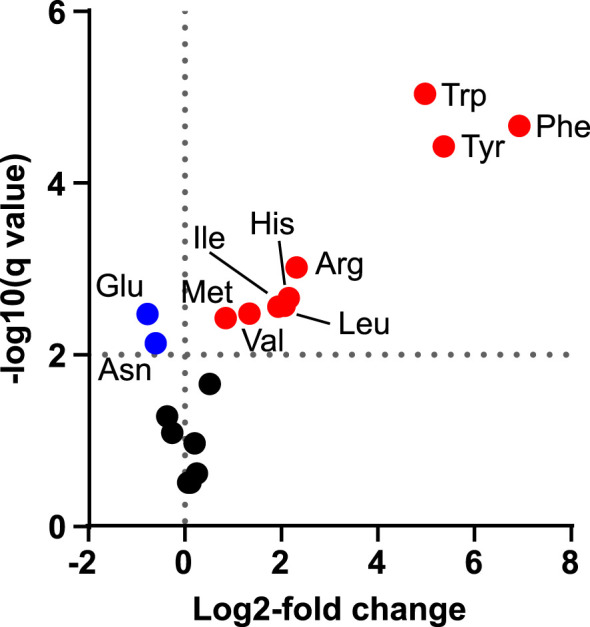
Changes in amino acid concentrations after transient expression of AroG*. Volcano plot shows the log2-fold difference in absolute amino acid abundance between plants infiltrated with pEAQ-EV alone or with pEAQ-EV and pN-AroG* together at equal OD quantified 5 dpi. Statistical significance was determined by unpaired t-tests with correction for multiple comparisons using two-stage step-up method of Benjamini, Krieger and Yekuteli (Benjamini et al., 2006), with a false discovery cutoff of 1% (*q = 0.01*, horizontal dashed line). Significantly increased amino acids are plotted in red, significantly decreased amino acids are plotted in blue and amino acids for which no significant difference was found in black.

We next decided to investigate if indican production could be further improved by boosting supply of substrates to CYP2A_B49. Because the indole substrate for CYP2A_B49 derives from conversion of tryptophan by tnaA, we hypothesized that increasing aromatic amino acid supply might increase indican production in our system. We had previously observed increased MS peak areas belonging to Phe, Tyr and Trp (not shown) after transient expression of a feedback insensitive variant of 3-deoxy-D-arabino-heptulosonate 7-phosphate synthase, AroG^D146N/A202T^ (Ding et al., 2014) - denoted AroG* in the following. To quantify this increase, we expressed AroG* targeted to chloroplasts with the CTP from AtFd2 using our pN vector together with pEAQ-EV and performed absolute quantification of 18 amino acids (Ala, Arg, Asn, Asp, Glu, Gln, His, Ile, Leu, Lys, Met, Phe, Pro, Ser, Thr, Trp, Tyr, Val) at 5 dpi. Plants expressing AroG* accumulated significantly more aromatic amino acids, with 115-, 49- and 37-fold increased accumulation of Phe, Tyr and Trp, respectively (**Figure 3**, **Table 1**). Expression of AroG* also caused significantly increased accumulation of Met, Val, Ile, Leu, His and Arg, but decreased Asp and Glu accumulation, albeit to a lesser extent (**Figure 3**, **Supplementary Table S3**). Total amino acid accumulation roughly doubled upon expression of AroG* compared to empty vector controls (**Supplementary Table S3**).

**Table 1:** Average absolute quantities and ratios of aromatic amino acids in tobacco plants (*n=7* leaves from individual plants per condition) after transient expression of pEAQ-EV or pEAQ-EV + pN-AroG* constructs. The asterisk is part of the name used for the aroG* gene in this study.

Amino acid	pEAQ-EV	pEAQ-EV + pN-AroG*	Ratio (AroG*/EV)
	*nmol gFW^-1^*		
Phenylalanine	12.0	1374.0	114.8
Tyrosine	5.8	286.3	49.4
Tryptophan	0.6	21.3	36.7

### Increased Trp or redox partner supply does not improve transient indican production

**Figure 4:**
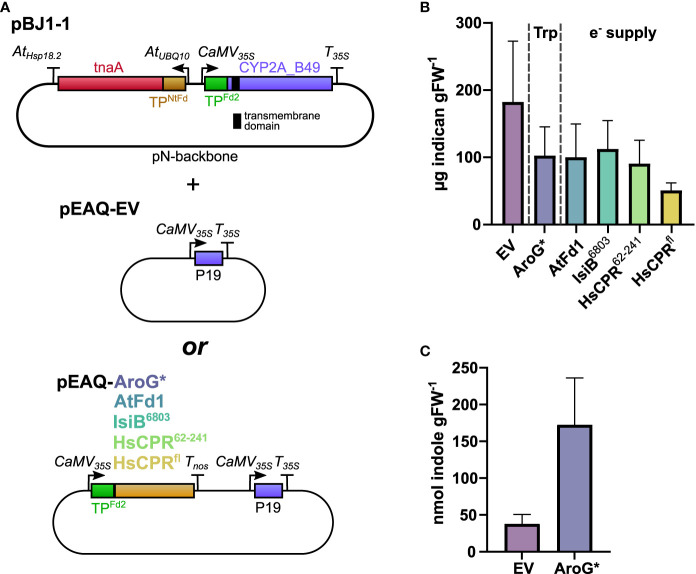
**(A)** Schematic of vector combinations used in this experiment. The transmembrane domain of CYP2A_B49 is indicated by a black bar. pBJ1-1 vector was co-infiltrated with either pEAQ-EV or a pEAQ containing AroG* or one of four redox partners. **(B)** Indican productivity upon co-infiltrating pBJ1-1 with pEAQ constructs carrying different transgenes. Two independent transient expression experiments were performed, and plants analyzed for indican at 5 dpi. Bars show average indican accumulation (*n* = 8 infiltrated leaves from individual plants per construct combination) with error bars indicating SD. **(C)** Indole concentrations quantified at 5 dpi in extracts from tobacco plants infiltrated with pBJ1-1 and either pEAQ-EV or pEAQ-AroG*. Bars show averages from a single experiment (*n = 5* infiltrated leaves from individual plants per construct combination)with error bars indicating standard deviation.

We wanted to test whether increasing Trp content would yield a corresponding increase in indican accumulation. An added benefit of the pBJ1-1 and -2 constructs is that we can co-express additional proteins by swapping them into the empty cloning site on the pEAQ-EV vector we co-infiltrate to deliver P19 (**Figure 4A**). This allows us to keep the total number of T-DNAs introduced constant and ensures consistent tnaA and CYP2A_B49 expression levels in each comparison. Upon co-infiltration of pEAQ-AroG* with pBJ1-1 we unexpectedly saw a drop in indican productivity compared to pBJ1-1 with pEAQ-EV (**Figure 4B**). To investigate whether this drop might be caused by an overall reduction in the activity of our pathway, we compared the accumulation of indole in our control (EV) and AroG* co-infiltrations by UPLC-UV. When co-infiltrating pBJ1-1 with AroG* we saw approximately four times more indole than with pEAQ-EV (**Figure 4C**). We also carried out untargeted MS analysis to investigate whether any other metabolite changes could explain the reduction in indican observed. Samples from co-expression of AroG* were clearly separated from EV control in PCA analysis (**Supplementary Figure S3A**). The main drivers of the separation were the aromatic amino acids as well as de-aminated and de-carboxylated Trp derivatives (**Supplementary Figure S3B**). Four compounds spectrally matched to 2-oxindoles mirrored the accumulation pattern observed for indican (**Figure 4B**, **Supplementary Figure S4**). Two of the features had retention times that coincided with indican, and probably arose from in-source fragmentation.

Next, we tested whether increasing the supply of reducing power to CYP2A_B49 might improve indican biosynthesis. We previously showed that it is possible to insert redox partners into the photosynthetic electron transfer chain at the photosystem I-stroma interface to alter the distribution of photosynthetically generated reducing power within the chloroplast. An engineered soluble flavodoxin-like protein derived from plant CPR could thus interact with and accept electrons directly from PSI (Mellor et al., 2019). To test whether the same could hold for our indican biosynthetic system, we introduced additional redox partners. As for AroG* co-expression, redox partner expression constructs were encoded on the pEAQ vector to avoid introducing additional T-DNAs into the infiltration mixture. We chose three soluble redox partners, namely AtFd1, which we have previously shown to be the ferredoxin best able to support light-driven activity of *Sorghum* CYP79A1 (Mellor et al., 2019), the cyanobacterial flavodoxin IsiB^6803^ from *Synechocystis* sp. PCC6803, and the flavodoxin-like domain from human CPR, HsCPR^62-241^, truncated to produce an independent soluble flavodoxin-like protein similar to the flavodoxin-like protein we previously generated from *Sorghum* CPR2b (Estrada et al., 2016; Mellor et al., 2019). We also wanted to test whether introducing the full-length CPR, and thus moving competition for reducing power from the level of ferredoxin to NADPH (**Figure 4A**) would improve overall activity. When we co-infiltrated pEAQ vector carrying either redox partner we saw a similar productivity as in AroG* co-expression, except for co-expression of HsCPR^fl^, in which case indican accumulation was about 25% that of the EV control (**Figure 4B**). These results show that productivity is not limited by availability of indole or redox supply, and that expressing additional transgenes from the pEAQ vector has detrimental effects on indican yield.

### *In vitro* comparison of ferredoxin and flavodoxin redox partners

To better understand how effectively different redox partners support CYP2A_B49 catalytic activity, we next expressed and purified the soluble redox carriers AtFd1, IsiB^6803^, and HsCPR^62-241^. All purified proteins showed UV-Visible spectra characteristic of their 2Fe-2S (AtFd1, **Supplementary Figure S4A**) or FMN cofactors (IsiB^6803^ and HsCPR^62-241^, **Supplementary Figure S4B**). Although both purified flavodoxins bound FMN, we performed isothermal titration calorimetry (ITC) to ensure that FMN binding was not affected by the truncation. Both flavodoxins exhibited native-like FMN binding (**Supplementary Figure S4**), with affinities (**Supplementary Table S4**) generally consistent with those reported for cyanobacterial flavodoxins and FMN-binding domains of eukaryotic CPR (Lian et al., 2011; Lans et al., 2012). Together these data show that truncated flavodoxin-like HsCPR^62-241^ retains the ability to bind FMN in a native-like manner.

**Figure 5:**
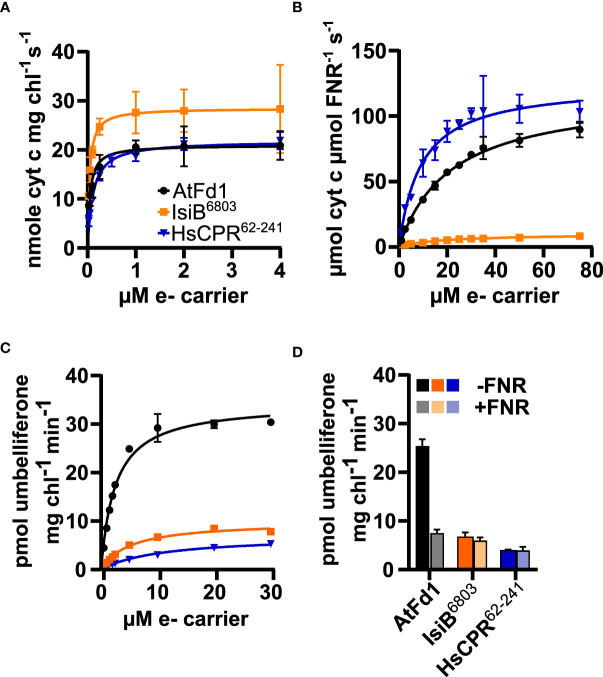
Flavodoxin and ferredoxin electron transfer kinetics with photosystem I **(A)**, FNR **(B)** and CYP2A_B49 **(C)**, and CYP2A_B49 + FNR competition assay **(D)**. **(A)** Kinetics of electron transfer from photosystem I to redox partners, measured by redox partner mediated cytochrome *c* reduction using isolated thylakoid membranes supplemented with varying concentrations of redox partners. **(B)** NADPH-dependent reduction of electron carrier proteins by FNR, measured by redox partner mediated cytochrome *c* reduction. **(C)** kinetics of photosynthesis-driven coumarin hydroxylation by CYP2A_B49 in isolated thylakoid membranes in the presence of different redox partners. D, comparison of coumarin hydroxylation rates from CYP2A_B49 with 10 µM redox partner in the presence or absence of 0.6 µM FNR and 1.6 mM NADP^+^. Plots show mean rates measured from three **(A, D)** or four **(B, C)** technical replicates with error bars indicating ± SD, as well as corresponding Michaelis-Menten fits **(A–C)**.

To examine the ability of purified redox partners to support photosynthesis-driven CYP2A_B49 activity we first measured the kinetics of electron transfer from photosystem I to each electron carrier. IsiB^6803^ outperformed the other electron carriers, with V_max_/K_M_ ratio (713) almost double that of AtFd1 (420), and nearly four times that of HsCPR^62-241^ (198), though all carriers showed sub-µM K_M_ values (**Figure 5A**, **Table 2**). We then assayed kinetics of NADPH-dependent reduction of electron carriers by FNR to investigate how strongly each electron carrier would interact with competing electron sinks present in chloroplasts. Unexpectedly, V_max_/K_M_ = 13.6 for HsCPR^62-241^ was about double that of AtFd1 (5.4), and 20-times higher than that of IsiB^6803^ (**Figure 5B**, **Table 2**). Finally, we measured steady state kinetics of CYP2A_B49 embedded in photosynthetic thylakoid membranes purified from tobacco after transient expression with each electron carrier protein. Because indoxyl rapidly oxidizes to form insoluble indigo dye *in vitro*, we instead adapted a commonly used surrogate assay for CYP2A6 activity that relies on 7-hydroxylation of coumarin into umbelliferone (Kim et al., 2005). This assay revealed that CYP2A_B49 was much more efficiently reduced by AtFd1 (V_max_/K_M_ = 13.8) compared to the flavodoxins (**Figure 5C**, **Table 2**), with 7 times higher catalytic efficiency than IsiB^6803^ (V_max_/K_M_ = 1.9) and 22 times higher catalytic efficiency than HsCPR^62-241^ V_max_/K_M_ = 0.6). Surprisingly, despite deriving from the native human CPR and reduced quite efficiently by photosystem I, HsCPR^62-241^ was the worst redox partner for light-driven CYP2A_B49 activity. To go further and investigate how the electron carrier proteins would support CYP2A_B49 activity in the presence of a competing electron sink measured its light-driven activity in the presence and absence of the competing enzyme FNR. FNR readily oxidizes ferredoxins and flavodoxins to reduce NADP^+^ to NADPH and consumes most of the photosynthetic reducing power in chloroplasts (Holfgrefe et al., 1997; Backhausen et al., 2000). Adding FNR and NADP^+^ in AtFd1-driven assays decreased CYP2A_B49 activity by about 80% compared with assays without FNR (**Figure 5D**). CYP2A_B49 activity was mostly unaffected in presence of FNR when flavodoxins were used to supply electron (**Figure 5D**). Despite the ability of IsiB^6803^ and HsCPR^62-241^ to support CYP2A_B49 activity with equal efficiency whether in the presence of FNR or not, CYP2A_B49 activity was still highest in the presence of AtFd1 even when subjected to competition from FNR.

**Table 2:** Average Michaelis-Menten parameters ( ± SD) of electron transfer reactions between photosystem I, FNR and CYP2A_B49 using the electron carrier proteins tested in this study.

	Photosystem I		FNR		CYP2A_B49	
Electron carrier	V_max_ _nmol *cyt c* mg chl_^-1^_s_^-1^	K_M_ _µM_	V_max_ _µmol cyt c µmol FNR_^-1^_s_^-1^	K_M_ _µM_	V_max_ _pmol umbelliferone mg chl_^-1^_min_^-1^	K_M_ _µM_
AtFd1	21.0 ± 0.8	0.05 ± 0.01	117.9 ± 3.4	21.7 ± 1.5	34.4 ± 0.7	2.5 ± 0.2
IsiB^6803^	28.5 ± 1.3	0.04 ± 0.01	10.0 ± 0.5	17.7 ± 2.3	9.9 ± 0.3	5.2 ± 0.4
HsCPR^62-241^	21.8 ± 0.5	0.11 ± 0.01	125.1 ± 4.9	9.2 ± 1.3	7.0 ± 0.5	11.0 ± 1.7

## Discussion

### A chloroplast targeted indican biosynthesis pathway is functional in tobacco

In this study we demonstrate expression and targeting of three human P450s to chloroplasts in tobacco (**Figure 1**) and investigate their light-dependent conversion of indole to the indigo precursor indican. Two of these P450s are natural human P450s, and the third a variant generated by DNA shuffling of three CYP2A isoforms. Functional expression of a mutant of human CYP2A6 targeted to chloroplast in tobacco was already demonstrated previously (Fräbel et al., 2018). While we did not control for differential accumulation of the three P450s compared, our results were generally in line with previous CYP2A6-CYP2E1 comparisons (Gillam et al., 1999; Warzecha et al., 2007). The present study underscores the applicability of the strategy and shows that photosynthesis can be used widely to drive P450 activity without co-expression of a dedicated reductase, because endogenous ferredoxin delivers reducing power to P450 enzymes with very high efficiency (**Figure 5**, **Table 2**). Stable expression of CYP2A6 with maize BX1 yielded 0.95 mg g_DW_^-1^ in 3–4-week-old plants (Warzecha et al., 2007), and was subsequently developed further by introducing bacterial tryptophan halogenases, which allowed synthesis of chloroindican at high yields (0.93 mg g_DW_^-1^) in a transient expression system (Fräbel et al., 2018). The titers reported here (~0.2 mg g_FW_^-1^) are approximately equal to that of plants stably expressing CYP2A6 (Warzecha et al., 2007) if accounting for the ~80% water content of tobacco leaf (Guo et al., 2019). This shows a remarkable ability of transient expression to produce high yields in shorter time than stable transfection. The speed and scalability of the transient expression system also makes it a valuable tool for fast turnover testing and may in some cases be preferred over stable transgenic plants for biomolecule production (Holtz et al., 2015; Schultz et al., 2019; Kaur et al., 2021). The data also shows that chloroplast targeting of the indican biosynthetic pathway is as viable a strategy as retaining the normal ER targeting of the P450. This is consistent with previous studies, which have shown that eukaryotic P450 enzymes are generally compatible with heterologous thylakoid membrane insertion (Nielsen et al., 2013; Xue et al., 2014; Gangl et al., 2015; Berepiki et al., 2016; Mellor et al., 2016; Li et al., 2019).

### Transient indican production in tobacco is limited by CYP2A_B49 activity

We investigate whether by boosting precursor supply or co-expressing redox partners could increase indican productivity. Expressing a feedback-insensitive DAHP synthase (Ding et al., 2014) yielded a nearly 37-fold increase in Trp accumulation and approximately 4-fold increase in indole accumulation (**Figure 3**, **Figure 4C**, **Table 1**). This accumulation is greater than previously reported for *N. tabacum* plants stably expressing feedback insensitive AroG^L175Q^, which yielded 43-, 24- and 10-fold increases in Phe, Tyr and Trp, respectively (Oliva et al., 2020). Despite the increased aromatic amino acid and indole accumulation, we did not observe an increase in indican accumulation when co-expressing AroG* (**Figure 4B**). We saw a puzzling decrease in indican accumulation when we expressed either AroG* or soluble redox partners from the pEAQ vector alongside the bi-directional pBJ1-1 vector encoding the indican pathway (**Figure 4**). We could not determine the exact reason for this decrease, but untargeted MS analysis did suggest new or unexplained products (**Supplementary Figure 3**). Possible explanations include effects due to re-use of the 35S promoter or the CTPs used to target enzymes to chloroplasts, either of which has implications for optimal construct design and should be investigate more thoroughly. We also cannot definitively rule out lower accumulation of CYP2A_B49 as a contributor to this difference. However, previous work has shown that while protein expression can be very different between separate batches of tobacco plants grown at different times (Buyel and Fischer, 2012), protein expression from a given construct within a batch is relatively consistent when infiltrated into leaves of the same age between plants when infiltrated into leaves of the same age between plants (Mellor et al., 2016). Given that these comparisons were always made together on the same batch of plants, and the only difference between infiltrations was the gene inserted in pEAQ vector, we consider reduced CYP2A_B49 expression a less likely explanation. Nevertheless, comparing AroG* and redox partner co-expression indicates that that CYP2A_B49 activity rather than supply of indole or redox equivalents poses the major bottleneck (**Figure 4B**). This is surprising, as it implies the enzyme is operating near its maximal rate in chloroplasts. The CYP2A_B49 variant used was selected from variant screening based on improved indican productivity in *E. coli*, but its actual kinetic parameters for indole hydroxylation have not been determined. CYP2A6, which performed very similarly in our initial P450 comparison (**Figure 1**) has k_cat_ and k_cat_/K_M_ values for indole hydroxylation of 0.62 s^-1^ and 2.4*10^3^ M^-1^ s^-1^, respectively (Zhang et al., 2009). This is 20 and 40 times less than average k_cat_ and k_cat_/K_M_ values surveyed from nearly 2000 enzymes (Bar-Even et al., 2011), and places CYP2A6 in the 16^th^ percentile of surveyed rates. For comparison, *Sorghum* CYP79A1 for which we previously demonstrated photosynthesis-dependent hydroxylation (Mellor et al., 2019) has a k_cat_ of 6.8 s^-1^ (Jensen et al., 2011), in the 42^nd^ percentile and near the average of enzymatic rates surveyed (Bar-Even et al., 2011). Altogether, these lines of evidence point to P450 activity as the major limiting factor for indican productivity in tobacco.

### Ferredoxin as a universal P450 redox partner for metabolic engineering

Our *in vitro* comparisons showed that AtFd1 coupled photosynthetic reducing power to CYP2A_B49 activity better than either IsiB^6803^ or HsCPR^62-241^ derived from the human CPR (**Figure 5C**). Unexpectedly, HsCPR^62-241^ had only 2-fold lower catalytic efficiency as a photosystem I electron acceptor than AtFd1, while IsiB^6803^ had higher efficiency than AtFd1 (**Figure 5A**, **Table 2**), which shows that both flavodoxins can integrate with photosynthetic electron transport. Instead, the differences in CYP2A_B49 activity probably arises by faster electro donation from ferredoxin to CYP2A_B49. When we assayed redox partner-dependent CYP2A_B49 activity in the presence of FNR, which competes for reduced ferredoxin and better reflects *in vivo* productivity (Yacoby et al., 2011; Nielsen et al., 2013; Mellor et al., 2016) activity driven by AtFd1 was severely limited (**Figure 5D**). On the other hand, CYP2A_B49 activity supported by either IsiB^6803^ or HsCPR^62-241^ was relatively unaffected (**Figure 5D**). HsCPR^62-241^ has a FMN redox potential of -246 mV at pH 7.5 (Das and Sligar, 2009), and for this redox partner the result is comparable to our previous finding for the flavodoxin-like domain of *Sorghum* CPR2b, whose redox potential of -267 mV (Mellor et al., 2019). Both FMN redox potentials are more positive than the standard redox potential of the NADP^+^/NADPH couple, the FAD cofactor of FNR (Pueyo et al., 1991) which helps render electron transfer thermodynamically unfavorable. IsiB^6803^ on the other hand possesses a redox potential close to that of ferredoxin by virtue of its role as a ferredoxin surrogate under iron-limiting conditions (Ferreira and Straus, 1994; Pierella Karlusich et al., 2014). As such, there should be no thermodynamic barrier to the passing of reducing power from IsiB^6803^ to FNR in our *in vitro* assays. We instead show that electron transfer between IsiB^6803^ and FNR is slow (**Figure 5B**), probably due to suboptimal interaction between them, which explains why presence of FNR does not affect IsiB^6803^-driven CYP2A_B49 activity.

Our data shows that ferredoxin provides optimal coupling of CYP2A_B49 to photosynthetic reducing power. Although coupling P450 activity to photosynthetic reducing power is still a relatively niche field, plant and cyanobacterial ferredoxins has been used as surrogate electron transfer partners in many reconstituted P450 systems (Wirtz et al., 2000; Jackson et al., 2002; Goñi et al., 2009; Jensen et al., 2012; Zhang et al., 2018; Mie et al., 2020; Zhu et al., 2020; Liu et al., 2022). Recently a survey of 16 microbial ferredoxins found ferredoxin from the cyanobacteria *Synechococcus elongatus* PCC 7942 to be the most efficient redox partner for 5 different P450s (Zhang et al., 2018). This was later expanded to include further 2 bacterial P450s favoring non-native cyanobacterial ferredoxin redox partners (Liu et al., 2022). Together with our own findings, these results suggest that plant and cyanobacterial [2Fe-2S] ferredoxins may be ideal generalist P450 redox partners. While this may seem surprising, many ancestral P450 redox partners were likely to have been either ferredoxins or flavodoxins (Paine et al., 2005), which implies the ability to interact with both redox partners could have been present early in the evolutionary history of this class of enzymes and was maintained after the rise of eukaryotes. At the same time ferredoxins have by virtue of their role as central redox distribution hubs been constrained by evolution to retain interactions with hundreds of enzymes (Hanke et al., 2011; Peden et al., 2013; Goss and Hanke, 2014). Such conservation of redox partner interactions in both P450s and ferredoxins explains why electron transfer complementarity was maintained across the evolutionary divide that confined these enzymes to different cellular compartments.

### Improving yields from photosynthesis-based indigo production

Overcoming the limitation on indican biosynthesis posed by the activity of CYP2A_B49 in the system described here can be approached either by increasing the expression of the P450 or by screening for variants or enzymes with higher activity. Increased accumulation of indican was obtained when encoding both CYP2A_B49 and tnaA genes on the same vector (**Figure 2**), but further improvements could be achieved by systematic optimization of construct design. Stably engineering the pathway for expression from the chloroplast genome may be the optimal strategy to reach maximal expression of enzymes for a given biosynthetic pathway in tobacco. Several reports have demonstrated high-level production of small molecules by introducing biosynthetic pathways into the chloroplast genome (Viitanen et al., 2004; Fuentes et al., 2016; Lu et al., 2017), and expression levels of individual proteins expressed from the chloroplast genomes have reached as high as 70% of total leaf protein (Ruhlman et al., 2010). Furthermore, the bacterial-like nature of the plastid genetic machinery has allowed the development of an inducible riboswitch-based T7 promoter system, which is highly advantageous for introduction of pathways whose activity cause deleterious effects on the host plant (Emadpour et al., 2015; Agrawal et al., 2022). Despite being an incredibly powerful approach towards plant-based biomanufacturing, generation of transplastomic plants is difficult and time consuming and far from routine in all but a few academic labs. In this regard, the scalability and ease of transient expression approaches allow them to remain viable alternatives towards high-level small molecule production (Reed et al., 2017).

Only a handful of P450s are currently known to perform indole hydroxylation at high rates. Early indole hydroxylating variants of P450 BM3 had K_M_ for indole at 2 mM, which meant that overall catalytic efficiency (1.4*10^3^ M^-1^ s^-1^) remained lower than those of human P450s (Li et al., 2008). Since, more recent BM3 variants showing improved rates (6.7 s^-1^) and K_M_ values (140 µM), and resultant much improved catalytic efficiency of 47.9*10^3^ M^-1^ s^-1^ (Rousseau et al., 2020). A bacterial CYP153 capable of hydroxylating indole was recently found to by screening homologs across bacterial genomes for conversion of indole to indigo (Fiorentini et al., 2018). This enzyme originates from an unclassified *Pseudomonas* accession (sp. 19-rlim) and is a soluble ferredoxin-dependent P450 whose kinetic characteristics have not been described. Both it and P450s BM3 variants may thus be interesting candidates for targeting to chloroplasts.

Several non-P450 enzymes, mostly deriving from prokaryotic indole metabolism, are known to catalyze formation of indigo. Bacterial flavin-containing monooxygenases (FMO) are NADPH-dependent enzymes, some of which have been found to produce indigo from indole (Choi et al., 2003; Ameria et al., 2015), and have been applied successfully in microbial indigo fermentation schemes (Han et al., 2011; Hsu et al., 2018). A flavin-dependent monooxygenase recently identified in the indican-producing plant *Polygonum tinctorium* (Inoue et al., 2021) could be the missing indole hydroxylase in the plant indican biosynthetic pathway. Targeting FMOs to chloroplasts would shift competition for reducing equivalents to NADPH instead of ferredoxin. Improved redox supply could be achieved in this system by engineering FMOs with higher NADPH affinity (Cahn et al., 2017; Jiang et al., 2020) or increasing NADPH supply (Assil-Companioni et al., 2020). Some bacterial naphthalene dioxygenases (NDO) and phenol hydroxylases (PHO) also hydroxylate indole (Fabara and Fraaije, 2020). The NDO are ferredoxin-dependent non-heme iron enzymes, while PHO obtain electrons from NADPH *via* a reductase comprising fused ferredoxin reductase-ferredoxin domains (Leahy et al., 2003) and as such both enzyme types could potentially be integrated directly with photosynthetic electron transport. NDO and PHO are however also heteromultimeric enzyme complexes dependent on multiple cofactors (Fabara and Fraaije, 2020), and their successful chloroplast targeting will require more complicated constructs designs. Establishing indigo production with these types of enzymes in plants warrants study, but their functional targeting and assembly in chloroplasts remains to be established.

This study shows the complexities one encounters when optimizing plant-based metabolic engineering to improve productivity. Though the tools and approaches in plant metabolic engineering grow increasingly sophisticated, the complex genetic and metabolic context found in plant hosts complicates engineering and makes highly parallelized experimental workflows difficult to implement. Systematic studies of genetic and metabolic pathway designs, and studies aimed at increasing the design toolbox would facilitate knowledge-based optimization in the future. In addition, integrating modelling, omics and targeted analytical workflows for accurate assessment of enzyme and metabolite levels, by now relatively routine in microbial metabolic engineering studies (Nielsen et al., 2022) would facilitate identification of pathway bottlenecks and propel green plant-based bioproduction platforms of the future.

## Conclusions

We demonstrate chloroplast-based production of indican from tryptophan using photosynthesis-P450 hydroxylation. We show that indican productivity can be improved by combining both tryptophanase and P450 on one T-DNA. Further, we boost tryptophan accumulation by co-expressing feedback insensitive mutant DAHP synthase, which increases indole but not indican yield. Co-expressing additional redox partners also leads to unaltered indican yield. Further *in vitro* investigation shows that ferredoxin provides optimal coupling of photosynthetic electron transport and P450 activity. Our results demonstrate that plant chloroplasts provide a viable membrane and redox environment for animal P450s. Thus, transient expression with chloroplast targeting can be used to generate active P450s that do not require additional redox partners and has applications for metabolic engineering or could be used to elucidate metabolite profiles of human P450s.

## Data availability statement

The raw data supporting the conclusions of this article will be made available by the authors, without undue reservation.

## Author contributions

SM, JB, and MP designed experiments. SM, JB, JI, and CC performed experimental work. SM, JB, CC, TL, EG, and MP analyzed the data. SM wrote the manuscript with help and feedback from JB, JI, CC, TL, EG, and MP. All authors contributed to the article and approved the submitted version.
