# Supplementary figures and images for: Exploiting photosynthesis-driven P450 activity to produce indican in tobacco chloroplasts
Source: Front Plant Sci. 2023 Jan 9;13:1049177. doi: 10.3389/fpls.2022.1049177 (PMC9890960; doi:10.3389/fpls.2022.1049177)

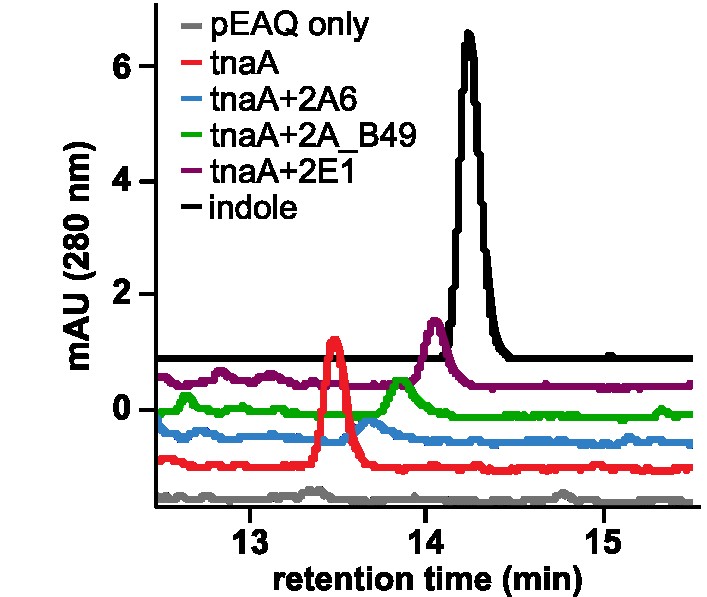

Supplement: Supplementary file 1 [file Image_1.jpeg]

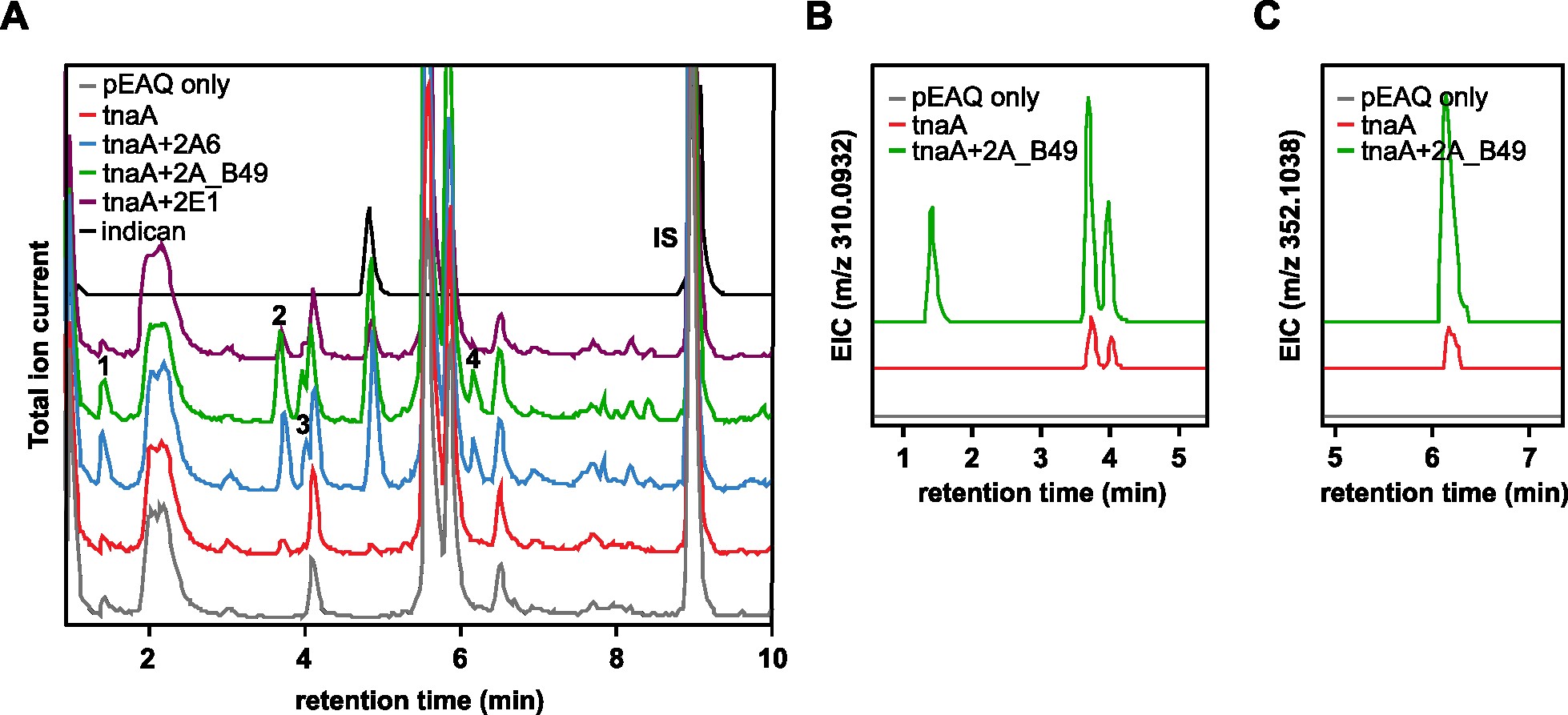

Supplement: Supplementary file 2 [file Image_2.jpeg]

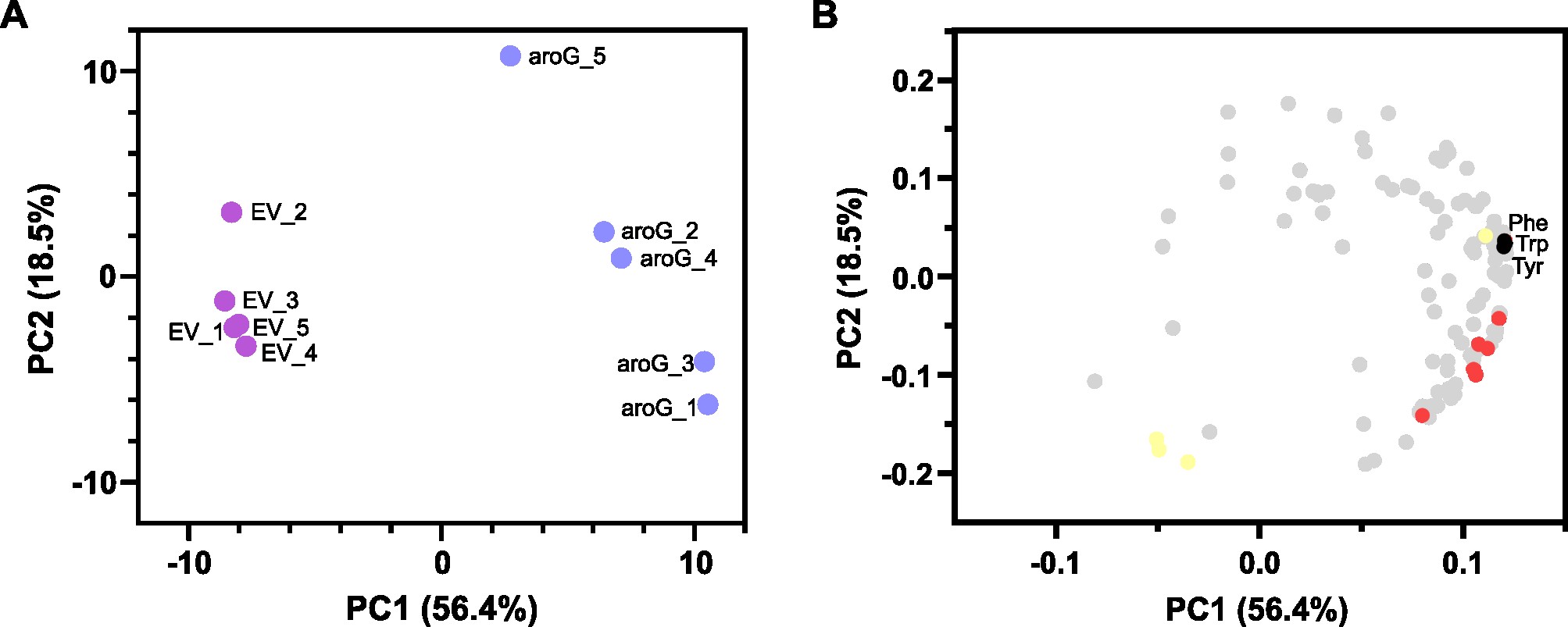

Supplement: Supplementary file 3 [file Image_3.jpeg]

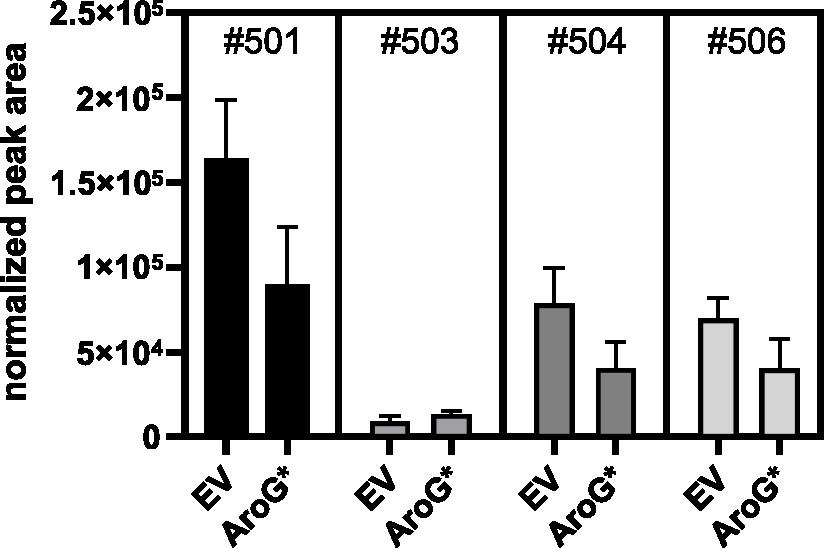

Supplement: Supplementary file 4 [file Image_4.jpeg]

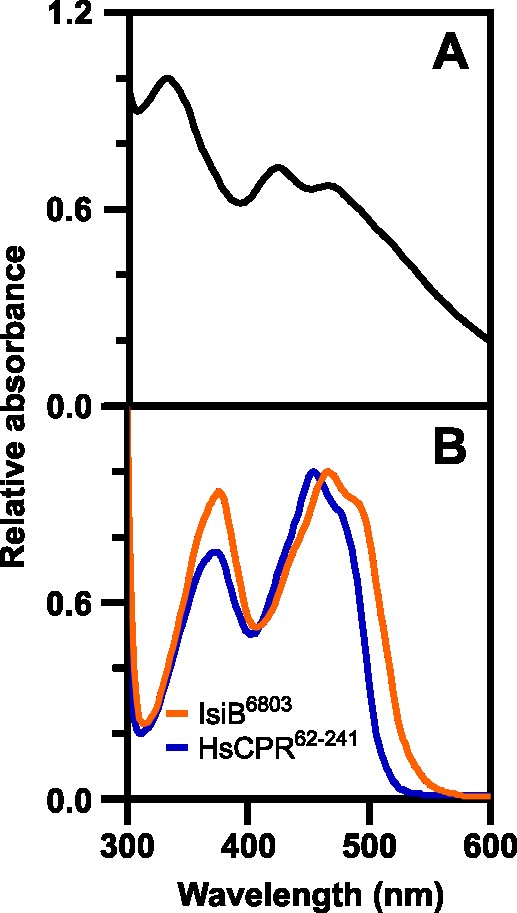

Supplement: Supplementary file 5 [file Image_5.jpeg]

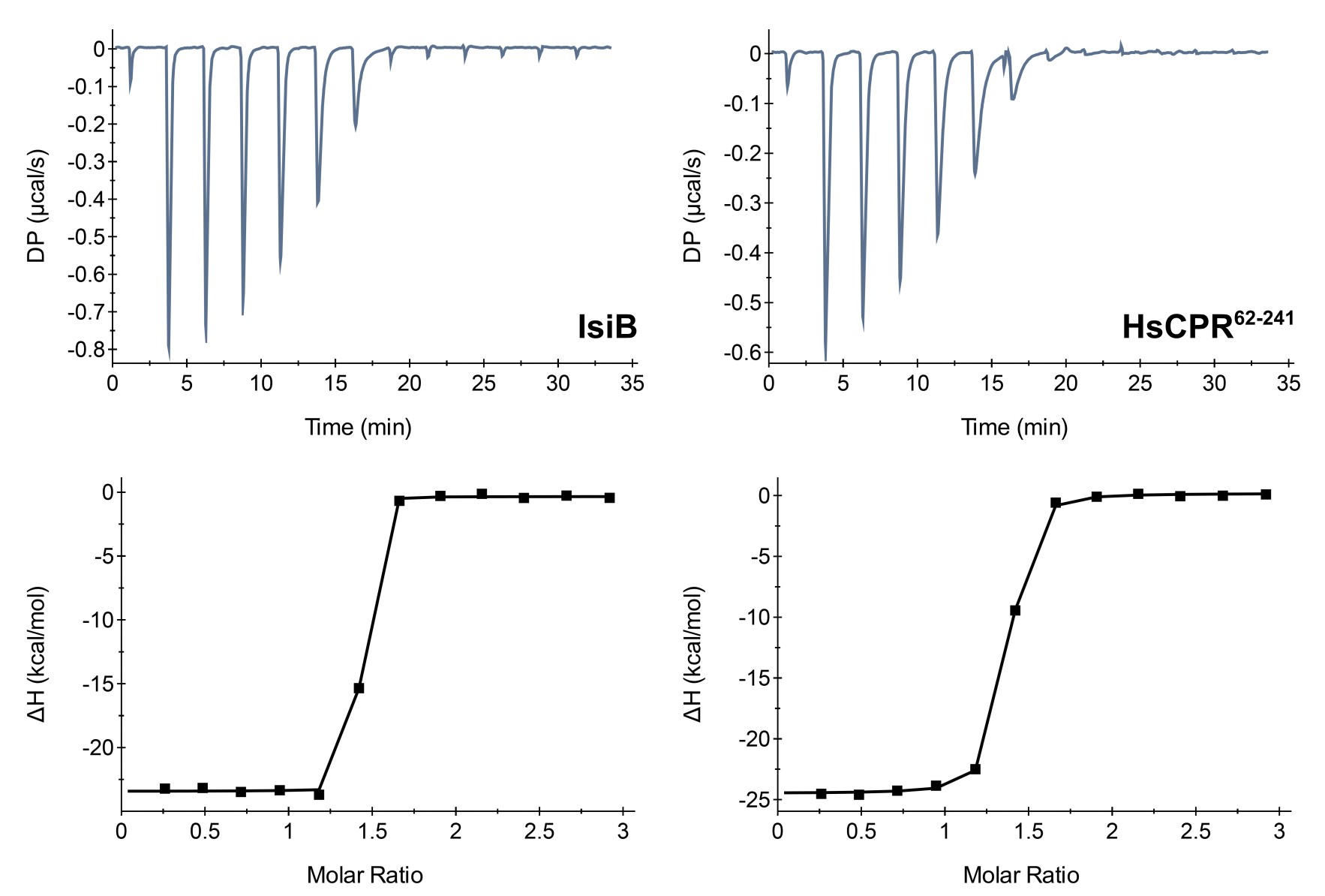

Supplement: Supplementary file 6 [file Image_6.jpeg]
