# Supplementary material for: Exploiting photosynthesis-driven P450 activity to produce indican in tobacco chloroplasts
Source: Front Plant Sci. 2023 Jan 9;13:1049177. doi: 10.3389/fpls.2022.1049177 (PMC9890960; doi:10.3389/fpls.2022.1049177)
Supplement: Supplementary file 7 [file Table_1.docx]

Supplementary Material

Supplementary Table S1. Nucleotide sequences for genes and genetic elements used in this study.

|  |  |  |  |
| --- | --- | --- | --- |
| *Gene* | Sequence | Constructs | Description |
| CYP2A6 | ATGGCTTCCACTGCTCTCTCAAGCGCCATCGTCGGAACTTCATTCATCCGTCGTTCCCCAGCTCCAATCAGTCTCCGTTCCCTTCCATCAGCCAACACACAATCCCTCTTCGGTCTCAAATCAGGCACCGCTCGTGGTGGACGTGTCACAGCCATGGCTGCTTCAGGAATGTTATTAGTGGCCTTGCTGGTCTGCCTGACTGTAATGGTCTTGATGTCTGTTTGGCAGCAGAGGAAGAGCAAGGGGAAGCTGCCTCCGGGACCCACCCCATTGCCCTTCATTGGAAACTACCTGCAGCTGAACACAGAGCAGATGTACAACTCCCTCATGAAGATCAGTGAGCGCTATGGCCCCGTGTTCACCATTCACTTGGGGCCCCGGCGGGTCGTGGTGCTGTGTGGACATGATGCCGTCAGGGAGGCTCTGGTGGACCAGGCTGAGGAGTTCAGCGGGCGAGGCGAGCAAGCCACCTTCGACTGGGTCTTCAAAGGCTATGGCGTGGTATTCAGCAACGGGGAGCGCGCCAAGCAGCTCCGGCGCTTCTCCATCGCCACCCTGCGGGACTTCGGGGTGGGCAAGCGAGGCATCGAGGAGCGCATCCAGGAGGAGGCGGGCTTCCTCATCGACGCCCTCCGGGGCACTGGCGGCGCCAATATCGATCCCACCTTCTTCCTGAGCCGCACAGTCTCCAATGTCATCAGCTCCATTGTCTTTGGGGACCGCTTTGACTATAAGGACAAAGAGTTCCTGTCACTGTTGCGCATGATGCTAGGAATCTTCCAGTTCACGTCAACCTCCACGGGGCAGCTCTATGAGATGTTCTCTTCGGTGATGAAACACCTGCCAGGACCACAGCAACAGGCCTTTCAGTTGCTGCAAGGGCTGGAGGACTTCATAGCCAAGAAGGTGGAGCACAACCAGCGCACGCTGGATCCCAATTCCCCACGGGACTTCATTGACTCCTTTCTCATCCGCATGCAGGAGGAGGAGAAGAACCCCAACACGGAGTTCTACTTGAAAAACCTGGTGATGACCACGTTGAACCTCTTCATTGGGGGCACCGAGACCGTCAGCACCACCCTGCGCTATGGCTTCTTGCTGCTCATGAAGCACCCAGAGGTGGAGGCCAAGGTCCATGAGGAGATTGACAGAGTGATCGGCAAGAACCGGCAGCCCAAGTTTGAGGACCGGGCCAAGATGCCCTACATGGAGGCAGTGATCCACGAGATCCAAAGATTTGGAGACGTGATCCCCATGAGTTTGGCCCGCAGAGTCAAAAAGGACACCAAGTTTCGGGATTTCTTCCTCCCTAAGGGCACCGAAGTGTACCCTATGCTGGGCTCTGTGCTGAGAGACCCCAGTTTCTTCTCCAACCCCCAGGACTTCAATCCCCAGCACTTCCTGAATGAGAAGGGGCAGTTTAAGAAGAGTGATGCTTTTGTGCCCTTTTCCATCGGAAAGCGGAACTGTTTCGGAGAAGGCCTGGCCAGAATGGAGCTCTTTCTCTTCTTCACCACCGTCATGCAGAACTTCCGCCTCAAGTCCTCCCAGTCACCTAAGGACATTGACGTGTCCCCCAAACACGTGGGCTTTGCCACGATCCCACGAAACTACACCATGAGCTTCCTGCCCCGCTGA | pN-CYP2A6 | Human CYP2A6 with transit peptide from *Arabidopsis thaliana* Fd2 |
| CYP2E1 | ATGGCTTCCACTGCTCTCTCAAGCGCCATCGTCGGAACTTCATTCATCCGTCGTTCCCCAGCTCCAATCAGTCTCCGTTCCCTTCCATCAGCCAACACACAATCCCTCTTCGGTCTCAAATCAGGCACCGCTCGTGGTGGACGTGTCACAGCCATGTCTGCCCTCGGAGTCACCGTGGCCCTGCTGGTGTGGGCGGCCTTCCTCCTGCTGGTGTCCATGTGGAGGCAGGTGCACAGCAGCTGGAATCTGCCCCCAGGCCCTTTCCCGCTTCCCATCATCGGGAACCTCTTCCAGTTGGAATTGAAGAATATTCCCAAGTCCTTCACCCGGTTGGCCCAGCGCTTCGGGCCGGTGTTCACGCTGTACGTGGGCTCGCAGCGCATGGTGGTGATGCACGGCTACAAGGCGGTGAAGGAAGCGCTGCTGGACTACAAGGACGAGTTCTCGGGCAGAGGCGACCTCCCCGCGTTCCATGCGCACAGGGACAGGGGAATCATTTTTAATAATGGACCTACCTGGAAGGACATCCGGCGGTTTTCCCTGACCACCCTCCGGAACTATGGGATGGGGAAACAGGGCAATGAGAGCCGGATCCAGAGGGAGGCCCACTTCCTGCTGGAAGCACTCAGGAAGACCCAAGGCCAGCCTTTCGACCCCACCTTCCTCATCGGCTGCGCGCCCTGCAACGTCATAGCCGACATCCTCTTCCGCAAGCATTTTGACTACAATGATGAGAAGTTTCTAAGGCTGATGTATTTGTTTAATGAGAACTTCCACCTACTCAGCACTCCCTGGCTCCAGCTTTACAATAATTTTCCCAGCTTTCTACACTACTTGCCTGGAAGCCACAGAAAAGTCATAAAAAATGTGGCTGAAGTAAAAGAGTATGTGTCTGAAAGGGTGAAGGAGCACCATCAATCTCTGGACCCCAACTGTCCCCGGGACCTCACCGACTGCCTGCTCGTGGAAATGGAGAAGGAAAAGCACAGTGCAGAGCGCTTGTACACAATGGACGGTATCACCGTGACTGTGGCCGACCTGTTCTTTGCGGGGACAGAGACCACCAGCACAACTCTGAGATATGGGCTCCTGATTCTCATGAAATACCCTGAGATCGAAGAGAAGCTCCATGAAGAAATTGACAGGGTGATTGGGCCAAGCCGAATCCCTGCCATCAAGGATAGGCAAGAGATGCCCTACATGGATGCTGTGGTGCATGAGATTCAGCGGTTCATCACCCTCGTGCCCTCCAACCTGCCCCATGAAGCAACCCGAGACACCATTTTCAGAGGATACCTCATCCCCAAGGGCACAGTCGTAGTGCCAACTCTGGACTCTGTTTTGTATGACAACCAAGAATTTCCTGATCCAGAAAAGTTTAAGCCAGAACACTTCCTGAATGAAAATGGAAAGTTCAAGTACAGTGACTATTTCAAGCCATTTTCCACAGGAAAACGAGTGTGTGCTGGAGAAGGCCTGGCTCGCATGGAGTTGTTTCTTTTGTTGTGTGCCATTTTGCAGCATTTTAATTTGAAGCCTCTCGTTGACCCAAAGGATATCGACCTCAGCCCTATACATATTGGGTTTGGCTGTATCCCACCACGTTACAAACTCTGTGTCATTCCCCGCTCATGA | pN-CYP2E1 | Human CYP2E1 with transit peptide from *Arabidopsis thaliana* Fd2 |
| CYP2A_B49 | ATGGCTTCCACTGCTCTCTCAAGCGCCATCGTCGGAACTTCATTCATCCGTCGTTCCCCAGCTCCAATCAGTCTCCGTTCCCTTCCATCAGCCAACACACAATCCCTCTTCGGTCTCAAATCAGGCACCGCTCGTGGTGGACGTGTCACAGCCATGTCTCTGATGTCCGTTTGGCGTCAGCGTAAATCCCGTGGTAAACTGCCGCCGGGTCCGACCCCGCTGCCGTTCATCGGTAACTACCTGCAGCTGAACACAGAGCAGATGTACAACTCCCTCATGAAGATCAGTGAGCGCTATGGCCCCGTGTTCACCATTCACTTGGGGCCCCGGCGGGTCGTGGTGCTGTGTGGACATGATGCCGTCAGGGAGGCTCTGGTGGACCAGGCTGAGGAGTTCAGCGGGCGGGGCGAGCAAGCTACCTTCGACTGGCTTTTCAAAGGCTATGGCGTAGTCTTCAGCAGCGGGGAGCGAGCCAAACAGCTAAGGCGCTTCTCCATCGCCACGCTGCGGGACTTCGGCGTGGGGAAGCGTGGCATCGAGGAGCGCATCCAAGAGGAGGCGGGCTTTCTCATCGATTCATTTCGGAAGACGAACGGTGCTTTTATTGACCCCACCTTCTACCTTAGCCGAACAGTCTCCAATGTCATTAGCTCAATTGTCTTCGGGGACCGCTTTGACTATGAGGACAAAGAGTTCCTGTCCCTGCTGCGTATGATGCTGGGTTCCTTCCAGTTCACCGCTACCTCCACCGGTCAGCTCTATGAGATGTTCTCTTCTGTGATGAAACACCTGCCAGGGCCCCAGCAACAGGCCTTTAAGGAGCTGCAGGGCCTGGAGGACTTCATAACCAAGAAAGTGGAACACAATCAGCGCACGCTGGATCCCAATTCCCCAAGGGACTTCATCGACTCCTTCCTCATCCGAATGCTGGAGGAGAAGAAGAACCCCAACACGGAGTTCTACTTGAAAAACCTGGTGATGACCACGTTGAACCTCTTCATTGGGGGCACCGAGACCGTCAGCACCACCCTGCGCTACGGTTTCCTGCTGCTCATGAAGCACCCAGAGGTGGAGGCCAAGGTCCATGAGGAGATTGACAGAGTGATCGGCAAGAACCGGCAGCCGAAATTCGAAGACCGTGCTAAAATGCCGTACACCGAAGCTGTTATCCACGAAATCCAGCGTTTCGGTGACATGCTGCCGATGGGTCTGGCTCACCGTGTTAACAAAGACACCAAATTCCGTGACTTCTTCCTGCCGAAAGGTACCGAAGTTTTCCCGATGCTGGGTTCCGTTCTGCGTGACCCGCGTTTCTTCTCCAACCCGCGTGACTTCAACCCGCAGCACTTCCTGGACAAAAAAGGTCAGTTCAAAAAATCCGACGCTTTCGTTCCGTTCTCCATCGGTAAACGTTACTGCTTCGGTGAAGGTCTGGCTCGTATGGAACTGTTCCTGTTCTTCACCACCATCATGCAGAACTTCCGTTTCAAATCCCCGCAGTCCCCGAAAGACATCGACGTTTCCCCGAAACACGTTGGTTTCGCTACCATCCCGCGTAACTACACCATGTCCTTCCTGCCGCGTGCGTCGACCCATCATCATCATCATCATTGA | pN-CYP2A_B49, pBJ1-1, pBJ1-2 | Engineered CYP2A_B49 with transit peptide from *Arabidopsis* *thaliana* Fd2 |
| tnaA(pN) | ATGGCTTCCACTGCTCTCTCAAGCGCCATCGTCGGAACTTCATTCATCCGTCGTTCCCCAGCTCCAATCAGTCTCCGTTCCCTTCCATCAGCCAACACACAATCCCTCTTCGGTCTCAAATCAGGCACCGCTCGTGGTGGACGTGTCACAGCCATGGAGAATTTTAAGCATTTACCTGAACCATTTCGTATTAGGGTCATTGAGCCTGTTAAACGAACCACCAGAGCATACAGGGAAGAAGCAATAATAAAATCCGGAATGAACCCTTTCTTGTTGGACAGTGAAGATGTGTTTATCGATTTGCTTACCGACTCCGGGACTGGAGCTGTCACTCAGTCAATGCAAGCCGCCATGATGAGGGGTGATGAGGCTTACTCAGGATCTAGATCCTACTACGCGCTGGCTGAATCTGTAAAGAATATTTTTGGGTATCAGTACACGATTCCAACGCACCAGGGCAGGGGCGCGGAACAAATTTATATCCCCGTTTTGATAAAAAAAAGGGAACAGGAAAAAGGTTTGGATAGAAGTAAGATGGTTGCATTCTCCAATTATTTTTTCGATACTACTCAGGGCCACAGCCAGATTAACGGTTGCACAGTCAGGAACGTCTACATTAAGGAGGCGTTCGATACTGGCGTACGATACGATTTCAAGGGGAACTTCGATCTTGAGGGGCTAGAACGTGGTATTGAAGAAGTTGGGCCCAATAATGTCCCCTACATTGTCGCCACGATAACGAGCAACTCTGCCGGAGGACAACCAGTTTCTTTAGCCAACCTCAAAGCGATGTATTCAATAGCGAAGAAGTATGATATTCCAGTAGTAATGGACTCAGCTCGATTCGCCGAGAACGCTTATTTTATCAAACAGAGGGAAGCCGAATATAAGGACTGGACCATTGAGCAGATAACCCGTGAGACCTATAAATATGCAGATATGCTTGCAATGTCTGCGAAGAAGGATGCAATGGTGCCTATGGGGGGACTATTATGTATGAAAGACGACAGTTTCTTTGATGTGTATACTGAGTGCAGGACTTTATGCGTAGTCCAAGAGGGCTTTCCCACCTACGGGGGTCTAGAAGGGGGTGCTATGGAGCGTCTAGCTGTTGGGCTTTACGACGGCATGAATCTGGACTGGTTGGCGTATAGAATCGCTCAGGTGCAATATCTCGTAGACGGTCTGGAGGAGATAGGGGTAGTCTGCCAACAGGCTGGCGGACATGCAGCGTTTGTTGACGCCGGGAAGCTCCTCCCGCACATACCTGCGGACCAGTTCCCAGCCCAGGCTTTGGCCTGTGAACTCTATAAAGTGGCTGGGATTCGTGCGGTTGAGATCGGATCTTTCTTACTCGGTAGAGACCCCAAGACTGGTAAGCAATTACCATGTCCAGCCGAGCTACTAAGATTGACGATTCCACGAGCGACATATACCCAAACTCATATGGACTTTATCATAGAAGCCTTTAAACATGTTAAAGAGAATGCCGCAAATATCAAGGGGCTCACGTTCACCTATGAACCAAAAGTGTTGAGGCATTTTACGGCAAAACTAAAAGAGGTCTAA | pN-tnaA | *E. coli* tryptophanase with transit peptide from *Arabidopsis* *thaliana* Fd2 |
| tnaA(pBJ) | ATGTCTACTATCAGCGTGCCCAGTGGTTGTTTCCTCAAGGTTGCACCATCTATTAGGAGCTCAGCCTTCACTAAGTCCCAGTGTTCATTAGGATCCGTCAAGTCCATATCTAAGGCTTTCGGGTTGAAATCCTCTTCCTCTTTCAAAGTTTCTGCAATGGAGAATTTTAAGCATTTACCTGAACCATTTCGTATTAGGGTCATTGAGCCTGTTAAACGAACCACCAGAGCATACAGGGAAGAAGCAATAATAAAATCCGGAATGAACCCTTTCTTGTTGGACAGTGAAGATGTGTTTATCGATTTGCTTACCGACTCCGGGACTGGAGCTGTCACTCAGTCAATGCAAGCCGCCATGATGAGGGGTGATGAGGCTTACTCAGGATCTAGATCCTACTACGCGCTGGCTGAATCTGTAAAGAATATTTTTGGGTATCAGTACACGATTCCAACGCACCAGGGCAGGGGCGCGGAACAAATTTATATCCCCGTTTTGATAAAAAAAAGGGAACAGGAAAAAGGTTTGGATAGAAGTAAGATGGTTGCATTCTCCAATTATTTTTTCGATACTACTCAGGGCCACAGCCAGATTAACGGTTGCACAGTCAGGAACGTCTACATTAAGGAGGCGTTCGATACTGGCGTACGATACGATTTCAAGGGGAACTTCGATCTTGAGGGGCTAGAACGTGGTATTGAAGAAGTTGGGCCCAATAATGTCCCCTACATTGTCGCCACGATAACGAGCAACTCTGCCGGAGGACAACCAGTTTCTTTAGCCAACCTCAAAGCGATGTATTCAATAGCGAAGAAGTATGATATTCCAGTAGTAATGGACTCAGCTCGATTCGCCGAGAACGCTTATTTTATCAAACAGAGGGAAGCCGAATATAAGGACTGGACCATTGAGCAGATAACCCGTGAGACCTATAAATATGCAGATATGCTTGCAATGTCTGCGAAGAAGGATGCAATGGTGCCTATGGGGGGACTATTATGTATGAAAGACGACAGTTTCTTTGATGTGTATACTGAGTGCAGGACTTTATGCGTAGTCCAAGAGGGCTTTCCCACCTACGGGGGTCTAGAAGGGGGTGCTATGGAGCGTCTAGCTGTTGGGCTTTACGACGGCATGAATCTGGACTGGTTGGCGTATAGAATCGCTCAGGTGCAATATCTCGTAGACGGTCTGGAGGAGATAGGGGTAGTCTGCCAACAGGCTGGCGGACATGCAGCGTTTGTTGACGCCGGGAAGCTCCTCCCGCACATACCTGCGGACCAGTTCCCAGCCCAGGCTTTGGCCTGTGAACTCTATAAAGTGGCTGGGATTCGTGCGGTTGAGATCGGATCTTTCTTACTCGGTAGAGACCCCAAGACTGGTAAGCAATTACCATGTCCAGCCGAGCTACTAAGATTGACGATTCCACGAGCGACATATACCCAAACTCATATGGACTTTATCATAGAAGCCTTTAAACATGTTAAAGAGAATGCCGCAAATATCAAGGGGCTCACGTTCACCTATGAACCAAAAGTGTTGAGGCATTTTACGGCAAAACTAAAAGAGGTCTAA | pBJ1-1, pBJ1-2 | *E. coli* tryptophanase with transit peptide from *Nicotiana tabacum* Fd |
| AroG* | ATGGCTTCCACTGCTCTCTCAAGCGCCATCGTCGGAACTTCATTCATCCGTCGTTCCCCAGCTCCAATCAGTCTCCGTTCCCTTCCATCAGCCAACACACAATCCCTCTTCGGTCTCAAATCAGGCACCGCTCGTGGTGGACGTGTCACAGCCATGAATTACCAAAATGATGACCTGAGAATTAAAGAAATTAAAGAGTTACTGCCACCAGTCGCCTTACTGGAAAAGTTCCCAGCAACCGAGAATGCTGCGAATACTGTAGCACATGCAAGGAAAGCCATCCATAAAATTTTGAAGGGTAATGACGACCGTCTGCTTGTCGTAATCGGCCCCTGCAGTATTCACGACCCGGTGGCGGCCAAGGAATATGCTACTCGACTACTTGCCTTAAGAGAAGAACTGAAGGACGAATTAGAGATTGTCATGCGAGTGTATTTTGAAAAGCCCAGGACTACGGTGGGTTGGAAGGGACTAATCAACGATCCGCATATGGACAACAGTTTTCAGATAAACGATGGTCTAAGAATTGCTAGAAAATTACTACTCGATATTAATGATAGCGGCTTACCAGCGGCAGGAGAATTCCTTAACATGATAACGCCCCAGTACCTTGCCGATCTCATGTCATGGGGCGCTATTGGCGCACGAACAACGGAGTCACAGGTCCACAGGGAGTTGGCGAGTGGCCTTAGTTGTCCTGTCGGCTTTAAAAATGGGACAGACGGTACCATCAAAGTAGCAATTGACGCGATAAACACAGCCGGTGCTCCCCATTGTTTTCTTAGCGTCACTAAATGGGGCCATTCTGCTATTGTTAATACTTCAGGAAACGGTGACTGTCACATAATACTGAGGGGCGGAAAAGAACCGAATTATAGCGCAAAGCATGTAGCCGAGGTTAAAGAGGGATTAAACAAAGCAGGATTGCCAGCTCAAGTGATGATTGATTTTTCCCACGCGAATTCTTCAAAGCAGTTCAAGAAACAGATGGATGTGTGTGCCGATGTTTGCCAACAAATAGCAGGCGGTGAGAAGGCCATTATCGGAGTAATGGTTGAGTCTCACCTGGTCGAGGGGAACCAAAGTCTCGAGTCTGGCGAACCGCTTGCTTACGGAAAGAGCATCACAGACGCATGTATTGGGTGGGAGGACACCGATGCCCTCCTGAGACAGCTAGCCAATGCCGTAAAAGCCCGTCGTGGGTAA | pN-AroG*, pEAQ-AroG* | *E. coli* AroG^D146N/A202T^ double mutant with transit peptide from *Arabidopsis thaliana* Fd2 |
| AtFd1 | ATGGCTTCCACTGCTCTCTCCAGCGCAATCGTAAGCACCTCTTTCCTCCGCCGTCAACAGACACCAATCAGCCTCAGATCCCTCCCGTTTGCCAACACACAATCTCTCTTCGGCCTCAAATCTTCCACCGCTCGCGGCGGCCGCGTCACGGCCATGGCTACCTACAAGGTCAAGTTCATCACACCTGAGGGAGAACAAGAGGTCGAATGCGAAGAAGATGTCTACGTCCTCGACGCTGCTGAGGAAGCCGGACTCGACTTGCCCTACTCATGCCGTGCCGGTTCTTGCTCAAGTTGCGCCGGGAAAGTCGTCTCTGGTTCTATTGACCAGTCGGACCAGAGCTTCTTAGACGATGAACAGATGAGTGAGGGCTATGTCTTGACCTGTGTGGCTTATCCGACTTCTGATGTCGTCATCGAAACCCACAAAGAAGAAGCCATTATGTAA | pEAQ-AtFd1 | *Arabidopsis thaliana* ferredoxin 1 with its native transit peptide |
| IsiB^6803^ | ATGGCTTCCACTGCTCTCTCAAGCGCCATCGTCGGAACTTCATTCATCCGTCGTTCCCCAGCTCCAATCAGTCTCCGTTCCCTTCCATCAGCCAACACACAATCCCTCTTCGGTCTCAAATCAGGCACCGCTCGTGGTGGACGTGTCACAGCCACAAAAATTGGACTTTTTTACGGTACTCAAACCGGCAACACTGAAACCATTGCTGAACTGATTCAAAAAGAAATGGGCGGCGATAGTGTGGTCGATATGATGGATATATCCCAGGCTGATGTTGATGATTTTAGGCAATATAGTTGCCTGATTATCGGTTGTCCCACCTGGAATGTGGGGGAACTCCAGAGTGATTGGGAAGGCTTTTATGACCAATTAGACGAAATTGATTTTAATGGCAAAAAAGTAGCCTATTTTGGTGCTGGCGATCAGGTTGGTTATGCAGATAATTTTCAAGACGCCATGGGCATTTTAGAAGAAAAAATCAGTGGATTAGGCGGTAAAACAGTGGGGTTTTGGCCCACCGCTGGCTATGATTTTGACGAATCAAAAGCGGTGAAAAATGGGAAATTTGTTGGTTTAGCTTTGGACGAAGATAATCAGCCAGAGTTAACAGAATTAAGAGTAAAGACATGGGTAAGTGAAATTAAACCAATTTTGCAATCCTAG | pEAQ-IsiB6803 | *Synechocystis* sp*.* PCC 6803 flavodoxin IsiB with transit peptide from *Arabidopsis* *thaliana* Fd2 |
| HsCPR^62-241^ | ATGGCTTCCACTGCTCTCTCAAGCGCCATCGTCGGAACTTCATTCATCCGTCGTTCCCCAGCTCCAATCAGTCTCCGTTCCCTTCCATCAGCCAACACACAATCCCTCTTCGGTCTCAAATCAGGCACCGCTCGTGGTGGACGTGTCACAGCCACATTGACCTCCTCTGTCAGAGAGAGCAGCTTTGTGGAAAAGATGAAGAAAACGGGGAGGAACATCATCGTGTTCTACGGCTCCCAGACGGGGACTGCAGAGGAGTTTGCCAACCGCCTGTCCAAGGACGCCCACCGCTACGGGATGCGAGGCATGTCAGCGGACCCTGAGGAGTATGACCTGGCCGACCTGAGCAGCCTGCCAGAGATCGACAACGCCCTGGTGGTTTTCTGCATGGCCACCTACGGTGAGGGAGACCCCACCGACAATGCCCAGGACTTCTACGACTGGCTGCAGGAGACAGACGTGGATCTCTCTGGGGTCAAGTTCGCGGTGTTTGGTCTTGGGAACAAGACCTACGAGCACTTCAATGCCATGGGCAAGTACGTGGACAAGCGGCTGGAGCAGCTCGGCGCCCAGCGCATCTTTGAGCTGGGGTTGGGCGACGACGATGGGAACTTGGAGGAGGACTTCATCACCTGGCGAGAGCAGTTCTGGCCGGCCGTGTGTGAACACTTTGGGGTGGAAGCCACTGGCGAGTAA | pEAQ-HsCPR^62-241^ | Human cytochrome P450 reductase FMN-binding domain comprising residues 62-241 with transit peptide from *Arabidopsis thaliana* Fd2 |
| HsCPR^fl^ | ATGGCTTCCACTGCTCTCTCAAGCGCCATCGTCGGAACTTCATTCATCCGTCGTTCCCCAGCTCCAATCAGTCTCCGTTCCCTTCCATCAGCCAACACACAATCCCTCTTCGGTCTCAAATCAGGCACCGCTCGTGGTGGACGTGTCACAGCCGCTGACTCCCACGTGGACACCAGCTCCACCGTGTCCGAGGCGGTGGCCGAAGAAGTATCTCTTTTCAGCATGACGGACATGATTCTGTTTTCGCTCATCGTGGGTCTCCTAACCTACTGGTTCCTCTTCAGAAAGAAAAAAGAAGAAGTCCCCGAGTTCACCAAAATTCAGACATTGACCTCCTCTGTCAGAGAGAGCAGCTTTGTGGAAAAGATGAAGAAAACGGGGAGGAACATCATCGTGTTCTACGGCTCCCAGACGGGGACTGCAGAGGAGTTTGCCAACCGCCTGTCCAAGGACGCCCACCGCTACGGGATGCGAGGCATGTCAGCGGACCCTGAGGAGTATGACCTGGCCGACCTGAGCAGCCTGCCAGAGATCGACAACGCCCTGGTGGTTTTCTGCATGGCCACCTACGGTGAGGGAGACCCCACCGACAATGCCCAGGACTTCTACGACTGGCTGCAGGAGACAGACGTGGATCTCTCTGGGGTCAAGTTCGCGGTGTTTGGTCTTGGGAACAAGACCTACGAGCACTTCAATGCCATGGGCAAGTACGTGGACAAGCGGCTGGAGCAGCTCGGCGCCCAGCGCATCTTTGAGCTGGGGTTGGGCGACGACGATGGGAACTTGGAGGAGGACTTCATCACCTGGCGAGAGCAGTTCTGGCCGGCCGTGTGTGAACACTTTGGGGTGGAAGCCACTGGCGAGGAGTCCAGCATTCGCCAGTACGAGCTTGTGGTCCACACCGACATAGATGCGGCCAAGGTGTACATGGGGGAGATGGGCCGGCTGAAGAGCTACGAGAACCAGAAGCCCCCCTTTGATGCCAAGAATCCGTTCCTGGCTGCAGTCACCACCAACCGGAAGCTGAACCAGGGAACCGAGCGCCACCTCATGCACCTGGAATTGGACATCTCGGACTCCAAAATCAGGTATGAATCTGGGGACCACGTGGCTGTGTACCCAGCCAACGACTCTGCTCTCGTCAACCAGCTGGGCAAAATCCTGGGTGCCGACCTGGACGTCGTCATGTCCCTGAACAACCTGGATGAGGAGTCCAACAAGAAGCACCCATTCCCGTGCCCTACGTCCTACCGCACGGCCCTCACCTACTACCTGGACATCACCAACCCGCCGCGTACCAACGTGCTGTACGAGCTGGCGCAGTACGCCTCGGAGCCCTCGGAGCAGGAGCTGCTGCGCAAGATGGCCTCCTCCTCCGGCGAGGGCAAGGAGCTGTACCTGAGCTGGGTGGTGGAGGCCCGGAGGCACATCCTGGCCATCCTGCAGGACTGCCCGTCCCTGCGGCCCCCCATCGACCACCTGTGTGAGCTGCTGCCGCGCCTGCAGGCCCGCTACTACTCCATCGCCTCATCCTCCAAGGTCCACCCCAACTCTGTGCACATCTGTGCGGTGGTTGTGGAGTACGAGACCAAGGCCGGCCGCATCAACAAGGGCGTGGCCACCAACTGGCTGCGGGCCAAGGAGCCTGCCGGGGAGAACGGCGGCCGTGCGCTGGTGCCCATGTTCGTGCGCAAGTCCCAGTTCCGCCTGCCCTTCAAGGCCACCACGCCTGTCATCATGGTGGGCCCCGGCACCGGGGTGGCACCCTTCATAGGCTTCATCCAGGAGCGGGCCTGGCTGCGACAGCAGGGCAAGGAGGTGGGGGAGACGCTGCTGTACTACGGCTGCCGCCGCTCGGATGAGGACTACCTGTACCGGGAGGAGCTGGCGCAGTTCCACAGGGACGGTGCGCTCACCCAGCTCAACGTGGCCTTCTCCCGGGAGCAGTCCCACAAGGTCTACGTCCAGCACCTGCTAAAGCAAGACCGAGAGCACCTGTGGAAGTTGATCGAAGGCGGTGCCCACATCTACGTCTGTGGGGATGCACGGAACATGGCCAGGGATGTGCAGAACACCTTCTACGACATCGTGGCTGAGCTCGGGGCCATGGAGCACGCGCAGGCGGTGGACTACATCAAGAAACTGATGACCAAGGGCCGCTACTCCCTGGACGTGTGGAGCTAA | pEAQ-HsCPR^fl^ | Full-length human cytochrome P450 reductase with transit peptide from *Arabidopsis thaliana* Fd2 |
|  |  |  |  |
| *Promoters and terminators* | |  |  |
| At_UBQ10_ | GTCGACGAGTCAGTAATAAACGGCGTCAAAGTGGTTGCAGCCGGCACACACGAGTCGTGTTTATCAACTCAAAGCACAAATACTTTTCCTCAACCTAAAAATAAGGCAATTAGCCAAAAACAACTTTGCGTGTAAACAACGCTCAATACACGTGTCATTTTATTATTAGCTATTGCTTCACCGCCTTAGCTTTCTCGTGACCTAGTCGTCCTCGTCTTTTCTTCTTCTTCTTCTATAAAACAATACCCAAAGAGCTCTTCTTCTTCACAATTCAGATTTCAATTTCTCAAAATCTTAAAAACTTTCTCTCAATTCTCTCTACCGTGATCAAGGTAAATTTCTGTGTTCCTTATTCTCTCAAAATCTTCGATTTTGTTTTCGTTCGATCCCAATTTCGTATATGTTCTTTGGTTTAGATTCTGTTAATCTTAGATCGAAGACGATTTTCTGGGTTTGATCGTTAGATATCATCTTAATTCTCGATTAGGGTTTCATAGATATCATCCGATTTGTTCAAATAATTTGAGTTTTGTCGAATAATTACTCTTCGATTTGTGATTTCTATCTAGATCTGGTGTTAGTTTCTAGTTTGTGCGATCGAATTTGTCGATTAATCTGAGTTTTTCTGATTAACAG | pBJ1-1, pBJ1-2 | Ubiquitin 10 promoter from *Arabidopsis thaliana* |
| Nt_UBQ.U4_ | GGAGGCTAACTACGTTAGAGCGCTAACGAGAATACTTCATATACCGTATTTTTTACGATAATAATAATGTAATGTGAAATTGCTATCCAAAAGGCACCTAATTTTGTCCACCGTTCAAAGGAAAGGACAAGGAAGTAGTAGCGTGTAGGTTTGGTGCTGTACAAAATAAGCAAGACACGTGTTGCCTTATTATAGGATAATCCATAAGGCAATTTCGTCTTAAGTCGGCCATTGCACCTTTAAAAGGAGCCTCTTTGTTCCCAAAATCTTCATCCTTTGATTTCTCTATTCTCAATATCTCCTCAATTTTTCTCTAGTCTTCAAACACTTCTCAAGGTACATTAACTTCTTCTTTCTTTTTGTTCCTCTTATTTTATGCTACTTTTATTTAATTTCGATCTATATTTTTAGGATCTAAATACTCATTTTTGATTTGTTTAATCGCTCTGTATATATGCACCAAGTTGAAATTTTTGTAAGTTTATTTTGTTCGGTCTATATTTTAAGATCTGAAATACCCTTTACTGAGAAAAAAAAAACTCAACCTTGATTTTGTTGTACCTGGTTGAATTTGTTATTGTTGTGTATACAGTTAAAAAACTCAAGTCTTGATTTTATTGTTTCCCTTTTGTAGTTTGTATATACATAGAGCTGAATTGGTGTTCTAATTTTGGTTGATTTTTATGTATACAGTATAAAATCGATCTTAGTTTTGTTCATTGATTTGTATTTGCACAAAGTTGGAATTTTGCGTTTGTTATTTTGATGATTGAAACCTTTTCTGTATATACAG | pBJ1-1, pBJ1-2 | Ubiquitin 10 promoter from *Nicotiana tabacum* |
| At_HSP18.2_ | ATATGAAGATGAAGATGAAATATTTGGTGTGTCAAATAAAAAGCTTGTGTGCTTAAGTTTGTGTTTTTTTCTTGGCTTGTTGTGTTATGAATTTGTGGCTTTTTCTAATATTAAATGAATGTAAGATCTCATTATAATGAATAAACAAATGTTTCTATAATCCATTGTGAATGTTTTGTTGGATCTCTTCTGCAGCATATAACTACTGTATGTGCTATGGTATGGACTATGGAATATGATTAAAGATAAG | pBJ1-1, pBJ1-2 | Heat shock protein 18.2 terminator from *Arabidopsis thaliana* |

**Supplementary** **Table S2:** MRM transitions for indican analysis by LC-QqQ-MS/MS.

| **Analyte** | **Retention Time**  **[min]** | **Q1**  **[*m/z*]** | **Q3 [*m/z*]** | **Fragmentor**  **[V]** | **CE**  **[V]** |
| --- | --- | --- | --- | --- | --- |
| Indican [M-H]^-^ | 1.63 | 294.1 | 160.8^Qt^ | 83 | 4 |
| pOH-benzaldehyde [M-H]^-^ | 1.88 | 121.0 | 93.1^Qt^ | 83 | 28 |

Qt = quantifier ion, CE = collision energy

Supplementary Table S3. Average absolute amounts and ratios of aromatic amino acids in tobacco plants after transient expression of pEAQ-EV or pEAQ-EV + pN-AroG* construct. Concentrations measured on extracts from single leaves of similar age from individual plants (n=7 per condition).

|  | pEAQ-EV | pEAQ-EV + pN-AroG* | Ratio  (AroG*/EV) |
| --- | --- | --- | --- |
| Amino acid | *nmol gFW^-1^* | |  |
| Ala | 37.3 | 30.7 | 0.8 |
| Arg | 2.8 | 18.1 | 6.5 |
| Asn | 29.7 | 19.9 | 0.7 |
| Asp | 80.0 | 64.9 | 0.8 |
| Glu | 216.2 | 132.2 | 0.6 |
| Gln | 712.7 | 774.7 | 1.1 |
| His | 1.7 | 9.9 | 5.9 |
| Ile | 5.0 | 27.0 | 5.4 |
| Leu | 7.7 | 45.4 | 5.9 |
| Lys | 156.8 | 184.0 | 1.2 |
| Met | 1.0 | 1.8 | 1.9 |
| Phe | 12.0 | 1374.0 | 114.8 |
| Pro | 311.3 | 377.1 | 1.2 |
| Ser | 49.0 | 54.1 | 1.1 |
| Thr | 21.7 | 32.2 | 1.5 |
| Trp | 0.6 | 21.3 | 36.7 |
| Tyr | 5.8 | 286.3 | 49.4 |
| Val | 12.1 | 37.3 | 3.1 |
| Total amino acids | 1663.1 | 3491.0 |  |

Supplementary Table S4. Average thermodynamic parameters for binding of FMN to flavodoxins calculated from 3 technical replicate ITC titrations for each flavodoxin.

|  | *N* | *K_D_*  (×10^-9^ M) | *∆H*  (kcal mol^-1^) | *∆G*  (kcal mol^-1^) | *-T∆S*  (kcal mol^-1^) |
| --- | --- | --- | --- | --- | --- |
| IsiB^6803^ | 1.28 | 2.0 | -22.63 | -13.43 | 9.19 |
| HsCPR^62-241^ | 1.26 | 25.7 | -24.50 | -10.40 | 14.15 |

**Supplementary Figure S1.** UV 280 nm chromatograms from UPLC separation of tobacco samples extracted from plants infiltrated with tnaA and P450 transgene combinations as indicated.

**Supplementary Figure S2.** Base peak chromatograms from UPLC-QTOF MS analysis of tobacco infiltrated with different tnaA and P450 transgene combinations as indicated. Peaks for abundant unknown compounds specific to tnaA or tnaA and P450 samples are indicated with 1-4. B, Extracted ion chromatograms for m/z 310.0932 consistent with dioxindole glucoside (peaks 1-3) have m/z values consistent with dioxindole glucosides. C, Extracted ion chromatograms for *m/z* 352.1038 consistent with hydroxylated indole acetic acid glucoside (peak 4).

**Supplementary Figure S3.** Principal component analysis from untargeted QTOF-MS analysis (positive mode). Data was processed with MS-DIAL, log-transformed and auto-scaled before PCA. Only features for which MS-DIAL produced matches to the MONA positive mode MS spectral library (https://mona.fiehnlab.ucdavis.edu) were used to generate the PCA scores. A, Score plot showing the separation of EV and AroG* samples along the first principal component axis. B, Loading plot. Aromatic amino acids are colored black, tryptophan derivatives are red, and 2-oxindoles colored yellow. The remaining compounds are shown as grey dots.

**Supplementary Figure S4.** Peak areas from four features with spectral matches to 2-oxindoles. The ‘#’ prefix denotes feature number. #504 and #506 have nearly identical retention times that coincide with that of indican. Peak areas were normalized to internal standard peak areas. Bars show averages peak areas (n=5 per condition), with error bars showing standard deviations.

**Supplementary Figure S5.** UV-Visible spectra of purified electron carrier proteins. A, spectrum of purified AtFd1. B, spectra of purified IsiB^6803^ and HsCPR^62-241^. Spectra shown are normalized to the maximal absorbance in the visible range (330, 466 and 454 nm, respectively for AtFd1, IsiB^6803^ and HsCPR^62-241^).

**Supplementary Figure S6.** Representative thermograms and binding isotherms for titration of FMN into purified apoflavodoxins. Top, baseline subtracted thermograms from titrating 150 µM FMN into a 15 µM solution of each apoflavodoxin as indicated. Lower, binding isotherms and fits for the titrations shown above.
